# Synthesis of Molecular Organo‐f‐Element‐Polyphosphides with Nonclassical Divalent Lanthanide Precursors

**DOI:** 10.1002/anie.202503403

**Published:** 2025-05-19

**Authors:** David Frick, Elena Pross, Ralf Köppe, Peter W. Roesky

**Affiliations:** ^1^ Institute of Inorganic Chemistry Karlsruhe Institute of Technology (KIT) Kaiserstr. 12 76131 Karlsruhe Germany; ^2^ Institute of Nanotechnology (INT) Karlsruhe Institute of Technology (KIT) Kaiserstr. 12 76131 Karlsruhe Germany

**Keywords:** Lanthanide, Phosphorous, Polyphosphide, Rare earth, Zintl ions

## Abstract

The coordination and functionalization of white phosphorus (P_4_) by transition metal and main group element compounds have led to a great variety of polyphosphides. However, larger polyphosphides of the rare‐earth elements are elusive. In this study, we report the synthesis and full characterization of the largest polyphosphides of the f‐elements, [K(18‐crown‐6)]_2_[(Cp′′_2_Ln)_2_(*μ*_4_‐*ƞ* ^2^:*ƞ* ^2^:*ƞ* ^2^:*ƞ* ^2^P_14_)], which was isolated along with [K(18‐crown‐6)][(Cp′′_2_Ln)_2_(*μ*‐*ƞ* ^3^:*ƞ* ^3^P_3_)] (Ln = La, Ce, Cp′′ = 1,3‐bis(trimethylsilyl)cyclopentadienyl). These compounds were obtained by using nonclassical divalent compounds of the early lanthanides for the reduction of white phosphorus.

## Graphical abstract

Herein we show the synthesis and characterization of organo‐f‐element‐functionalized polyanionic compounds, including the triangular P_3_^3−^ and the P_14_^4−^ polyphosphide Zintl anion. The title compounds, which are the largest organo‐f‐element polyphosphides known to date, were obtained by reduction of white phosphorus with precursors of the early nonclassical divalent lanthanides. Both compounds were isolated as the products of the same reaction.

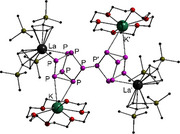

Zintl phases are intermetallic compounds consisting of alkali or alkaline earth metals and elements of groups 13–16 except N, O, and S.^[^ ^1^, ^2^ ^]^ Besides the formation of new structural motifs, recent work in Zintl chemistry also shed some light on the formation of nanomaterials.^[^ ^2^, ^3^, ^4^, ^5^, ^6^, ^7^, ^8^ ^]^ In the case of group 15 elements, the E_7_^3−^ (E = P, As, and Sb) nortricyclane‐type polypnictide anions are the most commonly known Zintl anions.^[^ ^2^, ^4^ ^]^ To obtain these species, reactions with the correct stoichiometry (3 K:7 E) must be carried out at high temperatures without solvent or in liquid ammonia.^[^ ^2^, ^5^ ^]^ In recent years, the study of complexes containing polyphosphides has made significant progress. One of the classical ways to obtain such compounds is via direct reduction and functionalization of white phosphorus (P_4_).^[^ ^9^, ^10^ ^]^ It is evident that there is a considerable level of interest in the coordination and functionalization of white phosphorus and metal polyphosphides.^[^ ^10^, ^11^, ^12^, ^13^, ^14^, ^15^ ^]^ Compared to the large amount of transition metal and main group element‐based polyphosphides, the amount of rare‐earth polyphosphides is still very limited.^[^ ^16^, ^17^, ^18^, ^19^, ^20^, ^21^, ^22^, ^23^, ^24^, ^25^, ^26^, ^27^ ^]^ In 2009, our group disclosed the first example of a rare‐earth polyphosphide, [{(Cp*)_2_Sm}_4_P_8_] (Cp* = *η* ^5^‐C_5_Me_5_), with a realgar‐type P_8_^4−^ structural motif via the reduction of white phosphorus with the highly reducing samarocene, [Sm(Cp*)_2_].^[^ ^28^ ^]^ Until today, this represents the largest rare‐earth polyphosphide, which can be obtained straight by reaction from P_4_. Direct functionalization of P_4_ with reactive rare‐earth compounds has also led to other smaller polyphosphide species with P_3_^3−^,^[^ ^16^ ^]^ P_4_^4−^,^[^ ^16^, ^19^, ^21^ ^]^ and P_7_^3−[^ ^18^, ^20^ ^]^ cores.

Another well‐established approach to obtain rare‐earth polyphosphides is the reaction with air‐stable transition‐metal polyphosphides^[^ ^29^ ^]^ such as pentaphosphaferrocene [(*η* ^5^‐P_5_)Fe(*η* ^5^‐Cp*)].^[^ ^1^, ^23^, ^24^, ^30^ ^]^ This iron sandwich complex contains a planar *cyclo*‐P_5_ ring, which can be further transformed to different polyphosphide anions.^[^ ^25^, ^29^, ^31^, ^32^, ^33^, ^34^ ^]^ In this context, the first P─P bond formation reactions between two [(*η* ^5^‐P_5_)Fe(*η* ^5^‐Cp*)] molecules triggered by divalent lanthanide complexes led to the largest polyphosphide of the lanthanides [(Cp*Fe)_2_P_10_{Sm(*η* ^5^‐C_5_Me_4_R)_2_}_2_] (R = Me, *n*Pr) until now.^[^ ^25^ ^]^ Recently, we have moved from the classical divalent lanthanides to the nonclassical divalent ones.^[^ ^18^, ^35^, ^36^, ^37^ ^]^ The discovery of these so‐called nonclassical divalent lanthanides (divalent lanthanides beyond Sm, Eu, and Yb) has opened another almost unexplored pathway toward unprecedented reactivity.^[^ ^38^, ^39^, ^40^, ^41^, ^42^ ^]^ The use of these compounds allowed, e.g., the isolation of the polyphosphide [K(18‐crown‐6)][Cp′′_2_La(P_5_)FeCp*] (Cp′′ = C_5_H_3_(SiMe_3_)_2_).^[^ ^18^ ^]^ Further reactions of these products with white phosphorus led to a selective expansion of the P_5_ rings to the nortricyclane‐type polyphosphide [K(18‐crown‐6)][Cp′′_2_La(P_7_)FeCp*].^[^ ^18^ ^]^

Herein, we report a pathway for the synthesis of the largest known organo‐f‐element polyphosphide, P_14_^4−^, by exploiting the high redox potential of nonclassical divalent compounds of the early lanthanides, namely the three‐ and four‐electron reductants [K(18‐crown‐6)][(Cp′′_2_La)_2_(*μ*‐*ƞ* ^6^:*ƞ* ^6^‐C_6_H_6_)] (**I**)^[^ ^41^, ^42^ ^]^ and [K(18‐crown‐6)]_2_[(Cp′′_2_Ce)_2_(*μ*‐*ƞ* ^6^:*ƞ* ^6^‐C_6_H_6_)] (**II**)^[^ ^18^ ^]^ (Figure 1).

**Figure 1:**
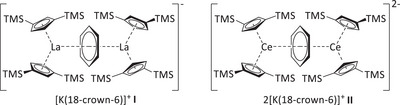
The three‐ and four‐electron reducing agents [K(18‐crown‐6)][(Cp′′_2_La)_2_(μ‐*ƞ* ^6^:*ƞ* ^6^C_6_H_6_)] and [K(18‐crown‐6)]_2_[(Cp′′_2_Ce)_2_(μ‐*ƞ* ^6^:*ƞ* ^6^C_6_H_6_)] featuring the nonclassical divalent lanthanides lanthanum and cerium (TMS = SiMe_3_).^[^ ^18^, ^41^, ^42^ ^]^

The literature known, strong multielectron reducing agents **I** and **II** (Figure 1), were used for the direct reduction of white phosphorus in THF to obtain organo‐f‐element polyphosphides. One equivalent of the respective reducing agent **I** or **II** was treated with 1.5 or 2 equivalents of P_4_, which resulted in the formation of the products [K(18‐crown‐6)]_2_[(Cp′′_2_Ln)_2_(*μ_4_*‐*ƞ* ^2^:*ƞ* ^2^:*ƞ* ^2^:*ƞ* ^2^‐P_14_)] (Ln = La (**1**), Ce (**2**)) in yields of 32% and 40%, respectively (Scheme 1). Suitable crystals of compounds **1** and **2** for single crystal X‐ray diffraction studies were obtained by slow evaporation of a solution of the respective compounds in toluene. While the tetradecaphosphides **1** and **2** were isolated by extraction of the reaction mixture with toluene, extraction of the reaction mixture with 1,2‐difluorobenzene allowed for the clean isolation of the triangular P_3_^3−^ polyphosphides [K(18‐crown‐6)][(Cp′′_2_Ln)_2_(*μ‐ƞ* ^3^:*ƞ* ^3^‐P_3_)] (Ln = La (**3**), Ce (**4**)) in yields of 23% for compound **3** and 12% for **4** of crystalline material (Scheme 1).

It is evident that the reactions of **I** and **II** with P_4_ exhibit significant disparities in comparison to those observed with nanoparticular arsenic (As_nano_). While at room temperature, the reaction of **I** with As_nano_ resulted in [{K(18‐crown‐6)}(Cp′′_2_La)_2_(*μ*_3_‐*ɳ* ^2^:*ɳ* ^2^:*ɳ* ^2^‐As_7_)], treatment of **II** with As_nano_ gave an inseparable mixture of an As_7_ and an As_14_ compound.^[^ ^43^ ^]^

Complexes **1** and **2** crystallize in the triclinic space group *P* $\bar{1}$ and are isostructural to each other. The molecular structures of both compounds in the solid state are depicted in Figure 2. The asymmetric unit contains two half molecules, subsequently only one of these will be discussed (details of the complete data are outlined in the Supporting Information). Both of these compounds, **1** and **2**, consist of a P_14_^4−^ tetradecaphosphide Zintl ion, which can be interpreted as a P─P bridged dimer of two nortricyclane P_7_^2−^ units, as well as two [K(18‐crown‐6)]^+^ and two [Cp′′_2_Ln]^+^ fragments. The P_14_^4−^ structural motif has only been reported previously for alkali metals, e.g., [Li(NH_3_)_4_]_4_P_14_ **·**(NH_3_),^[^ ^44^ ^]^ [Li_4_(THF)_10_]P_14_,^[^ ^45^ ^]^ Na_4_(en)_6_P_14_^[^ ^43^ ^]^ and Na_4_(DME)_7.5_P_14_.^[^ ^43^ ^]^ In the P_14_^4−^ unit of compounds **1** and **2**, the P─P bonds between the uncharged P4, P5, and P6 atoms are the longest and vary between 2.2238(9) and 2.2541(10) Å for the lanthanum compound **1**. In **2**, these bond lengths are between 2.233(2) and 2.243(2) Å. The P7─P7´ bonds act as a linkage between the two P_7_^2−^ units with bond lengths of 2.2343(13) Å in **1** and 2.228(2) Å in **2**. These bond lengths are in the same range as the other bonds between the uncharged phosphorus atoms. The bonds toward the charged phosphorus atoms (P1, P3) are shorter and in the range of 2.1557(10) and 2.1875(9) Å in **1** and vary between 2.159(2) and 2.190(2) Å in **2**. These P─P bond lengths are slightly longer compared to the known lithium salt [Li(NH_3_)_4_]_4_P_14_ **·**(NH_3_)^[^ ^44^ ^]^ with its bond lengths being between 2.122(2) and 2.176(2) Å. The sodium compound Na_4_(DME)_8_P_14_^[^ ^43^ ^]^ also shows shorter bond lengths, varying between 2.139 and 2.168 Å. In both cases, the potassium atoms in the [K(18‐crown‐6)]^+^ units coordinate to the polyphosphide via two phosphorus atoms. The distances between K1 and P4 or P6 stretch between 3.6694(10) and 3.6892(11) Å in **1** and between 3.4018(15) and 3.781(2) Å in **2**. Those bonds are much longer compared to the alkali metal tetradecaphosphides discovered prior^[^ ^43^, ^44^ ^]^ but in the same range as other organo‐f‐element polyphosphides.^[^ ^18^ ^]^ The lanthanide ions of both compounds are side‐on coordinated to the negatively charged phosphorus atoms (P2, P3) of the P_14_^4−^ Zintl ion. The La─P distances in compound **1** vary between 3.0615(7) and 3.0192(7) Å. As expected, due to the lanthanide contraction and the smaller ionic radius of Ce^3+^ compared to La^3+^, the Ce─P distances in compound **2** are slightly shorter, ranging from 2.9773(13) to 3.0353(13) Å. These distances are similar to those in other organo‐f‐element polyphosphides.^[^ ^18^, ^28^ ^]^

**Figure 2:**
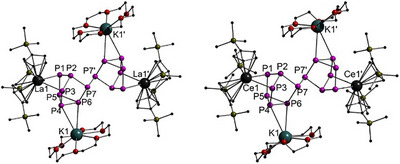
Molecular structures of [K(18‐crown‐6)]_2_[(Cp′′_2_La)_2_(*μ*_4_‐*ƞ* ^2^:*ƞ* ^2^:*ƞ* ^2^:*ƞ* ^2^P_14_)] (**1**) and [K(18‐crown‐6)]_2_[(Cp′′_2_Ce)_2_(*μ*_4_‐*ƞ* ^2^:*ƞ* ^2^:*ƞ* ^2^:*ƞ* ^2^P_14_)] (**2**) in the solid state. Hydrogen atoms, solvent molecules, and second [K(18‐crown‐6)]_2_[(Cp′′_2_Ln)_2_(*μ*_4_‐*ƞ* ^2^:*ƞ* ^2^:*ƞ* ^2^:*ƞ* ^2^P_14_)] are omitted for clarity. Only one part of the positional disorder of all four [K(18‐crown‐6)]^+^ is shown. For bond lengths, angles, and full picture of both molecules, see Supporting Information.

**Scheme 1:**
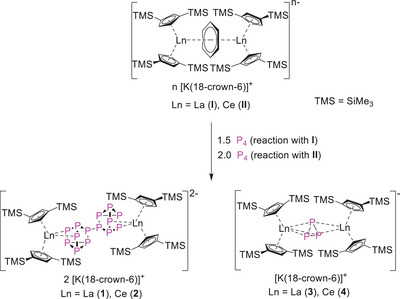
Synthesis of [{K(18‐crown‐6)}_2_(Cp′′_2_Ln)_2_(P_14_)] and [{K(18‐crown‐6)}(Cp′′_2_Ln)_2_(P_3_)] (Ln = La (**1**, **3**) and Ce (**2**, **4**); *n* = 1 for **I**, 2 for **II**).

Compounds **1** and **2** are only stable in solution and decompose rapidly when the solvent is removed. Thus, in order to obtain meaningful NMR spectra of the bulk material, the solvents used during the extraction and washing steps were not completely removed under reduced pressure. This results in signals of the remaining solvents in the ^1^H and ^13^C NMR spectra. Due to the paramagnetic behavior of the cerium complexes (**2** and **4**), no meaningful ^1^H and ^13^C NMR spectra could be obtained. The ^1^H NMR spectrum of **1** shows the four expected signals of the product. The singlet at *δ* = 0.08 ppm represents the hydrogen atoms of the TMS groups and the multiplets at *δ* = 6.00–6.03 ppm and *δ* = 6.09–6.11 ppm are assigned to the aromatic protons of the Cp′′ ligand. Up‐ and downfield shifts to the TMS group signal are the signals of the TMS groups of the decomposition product. The singlet at *δ* = 3.58 ppm represents the hydrogen atoms of the 18‐crown‐6 crown ether. As observed earlier for the P_14_^4−^ unit in Na_4_(en)_6_P_14_^[^ ^43^ ^]^ the signals in the ^31^P{^1^H} NMR spectrum could not be assigned to specific phosphorus atoms due to the dynamic behavior of the system. Furthermore, a partial decomposition of the product, resulting in the formation of different phosphorus‐containing species, was observed. A ^31^P{^1^H} NMR at 193 K was measured in an attempt to obtain a more defined spectrum; however, it was still not possible to assign the signals to specific phosphorus atoms. It was tried to measure a ^31^P COSY NMR spectrum to understand the phosphorus interaction, but the solubility of compound **1** is not high enough to get a reasonable spectrum with medium scan numbers. The ^29^Si{^1^H} NMR spectrum of **1** shows three signals, one singlet at *δ* = −14.6 ppm, which represents the TMS groups of the Cp′′ ligand, and the singlets at *δ* = −11.82 ppm and *δ* = −2.09 ppm belong to the decomposition products.

The complexes [K(18‐crown‐6)][(Cp′′_2_La)_2_(*μ*‐*ƞ* ^3^:*ƞ* ^3^P_3_)] (**3**) and [K(18‐crown‐6)][(Cp′′_2_Ce)_2_(*μ*‐*ƞ* ^3^:*ƞ* ^3^P_3_)] (**4**) crystallize in the monoclinic space group *P*2_1_/*c* and are isostructural to each other. Their molecular structures are depicted in Figure 3. The structures consist of an almost ideal triangular P_3_^3−^ anion, which shows bond lengths ranging from 2.1833(8) and 2.1907(8) Å in **3**, and between 2.1782(10) and 2.1888(10) Å in **4**. These bond lengths are in the same range as d‐metal complexes with coordinating P_3_^3−^ units, such as the complex [Na(OEt_2_)]_2_[(*η* ^3^‐P_3_)Nb(N[Np]Ar)_3_]_2_ (Ar = 3,5‐Me_2_C_6_H_3_,Np = CH_2_ *^t^* Bu) with its bond lengths ranging between 2.1724(7) and 2.1749(7) Å.^[^ ^46^ ^]^ Other d‐metal complexes with inverse sandwich‐like structures, such as [Na(thf)_5_][{Na(thf)_2_}_2_{(O_3_C)Zr}_2_(*μ*‐cyclo‐P_3_)](O_3_C = [(3,5‐*^t^* Bu_2_‐2‐*O*−C_6_H_2_)_3_C]^4–^)^[^ ^47^ ^]^ or [K(thf)_6_][(LCo)_2_(*μ*_2_:*η* ^3^,*η* ^3^‐P_3_)]·2 thf (L = CH[C(Me)N(2,6‐*^i^* Pr_2_C_6_H_3_)]_2_) show slightly longer P─P bond lengths, ranging between 2.217(4) to 2.237(4) Å compared to compounds **3** and **4**.^[^ ^47^ ^]^ Prior reported lanthanide complexes with bridging P_3_^3−^ units have P─P bond lengths in the same range as **3** and **4**.^[^ ^16^, ^21^ ^]^ The angles within the phosphorus triangle in **3** range from 59.81(3)° to 60.14(3)° indicating near‐perfect equilateral triangular structure. In **4**, the angles vary between 59.71(4)° and 60.19(3)°. The bond lengths between the lanthanum and the phosphorus atoms are in the range of 2.9007(6) and 2.9443(6) Å. In compound **4**, the bond lengths vary between 2.8745(7) and 2.9190(7) Å. These are slightly longer than in established complexes in the literature.^[^ ^16^, ^21^ ^]^ The lanthanide atoms and the phosphorus triangle are not in line and are angled between 126.87(2)° and 131.33(2)° in **3** and in **4** between 126.53(2)° and 130.88(2)°. In **3**, the lanthanide atoms have a distance to the centroids of the Cp′′‐plane between 2.59708(8) and 2.61302(8) Å and angles between 118.335(5)° and 119.219(5)°. In the cerium compound **4**, the distances to the centroids vary between 2.57201(9) and 2.58067(9) Å. The angles between are 118.221(5)° and 119.092(5)°. As expected, for compound **4** containing the smaller cerium atom, the distances and angles are smaller.

**Figure 3:**
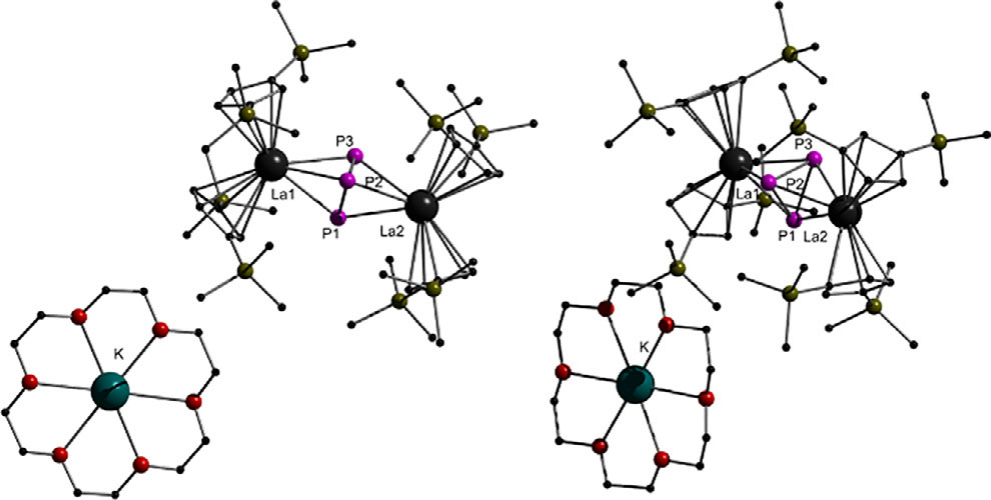
The molecular structure of [K(18‐crown‐6)][(Cp′′_2_La)_2_(*μ*‐*ƞ* ^3^:*ƞ* ^3^P_3_)](**3**) in two different perspectives in solid state. For clarity, all hydrogen atoms and solvent molecules are omitted. Full overview of bond lengths and angles is provided in the Supporting Information.

The ^31^P{^1^H} NMR spectrum of compound **3** shows one singlet at *δ* = −156.39 ppm, which indicates that the three phosphorus atoms are chemically equivalent in solution. The observed chemical shift is significantly different from Y‐*cyclo*‐P_3_‐Y compounds such as [{(*N*,*N*′‐2,6‐diisopropylphenyl‐1,4‐diazabutadiene)Y·THF}_2_{*cyclo*‐P_3_}K] (*δ* = −273.66 ppm)^[^ ^21^ ^]^ or [K{(NON)Y(thf)}_2_(*μ*_3_‐*η* ^3^:*η* ^3^:*η* ^2^‐P_3_)] (*δ* = −240.3 ppm) ((NON)^2−^ = 4,5‐bis(2,6‐diisopropylphenyl‐amino)‐2,7‐di‐*tert*‐butyl‐9,9‐dimethylxanthene).^[^ ^16^ ^]^ The observed difference may be a result of the different coordination of the potassium ion. While the K ion binds to the *cyclo*‐P_3_ unit in the latter compounds, **3** is an ionic compound in which the K ion is encapsulated by 18‐crown‐6 and thus no interaction with the *cyclo*‐P_3_ unit is observed. A similar behavior was previously observed for [Na(OEt_2_)]_2_[(*η* ^3^‐P_3_)Nb(N[Np]Ar)_3_]_2_, where the ^31^P{^1^H} NMR signal of the P_3_^3−^ anion shifts from *δ* = −223 ppm to *δ* = −183 ppm upon complexation with 12‐crown‐4 of the sodium cation.^[^ ^46^ ^]^ In other transition metal complexes, the chemical shifts range between *δ* = −185 ppm for molybdenum and *δ* = −230 ppm tungsten complexes.^[^ ^48^ ^]^ In contrast to that, inverse sandwich‐like complexes of nickel and cobalt were found to be ^31^P{^1^H} NMR silent.^[^ ^47^, ^49^, ^50^ ^]^ The ^1^H NMR spectrum of **3** shows the expected four signals representing the product. A singlet at *δ* = 0.25 ppm, which originates from the TMS groups and the multiplets at *δ* = 6.13–6.21 and *δ* = −6.64–6.69 ppm of the aromatic proton were observed. The singlet of the 18‐crown‐6 ether protons is located at *δ* = −3.61 ppm.

We have investigated the bonding situation using quantum chemical methods, in particular the P_14_^4−^ ligand to the La^3+^ metal center in the anionic fragment [(Cp′′_2_La)_2_(*μ*_4_‐*ƞ* ^2^:*ƞ* ^2^:*ƞ* ^2^:*ƞ* ^2^P_14_)]^2−^ of compound **1**. As stated before, P_14_^4−^ can be interpreted as the dimer of two P_7_ units. This model is confirmed by comparing the theoretical results of [(Cp′′_2_La)_2_(*μ*_4_‐*ƞ* ^2^:*ƞ* ^2^:*ƞ* ^2^:*ƞ* ^2^P_14_)]^2−^ with those of P_7_^3−^ whose bonding properties have been intensively investigated before by Zhai et al.^[^ ^51^ ^]^ P_7_^3−^ (symmetry *C_3v_*) is set up in the following manner (numbering of the atoms as given in the P_7_ subunit of **1** in Figure 2): The phosphorus atoms are sorted into three subgroups according to their coordination environments. P2 represents one apical P atom at the top of the P_7_ cage. Three atoms, P1, P3, and P7, are located on the waist of the cage, whereas three sites, P4–P6, form the base triangle. Specifically, P1, P3, and P7 are dicoordinated, being formally negatively charged. The partial charges Q according to a population analysis based on occupation numbers are determined to be −0.05 (P2), −0.69 (P1), or −0.30 (P4), respectively. The highest three occupied MOs (irreducible representations a_2_ or e) are primarily dominated by free electron pairs of p‐type of the atoms P1, P3, and P7. The shared electron numbers of the P─P bonds as a measure of covalent bond strength give rise to typical single bonds (SEN(P1─P2): 1.21, SEN(P4─P5): 0.92) or pronounced single bonds (SEN(P1─P4): 1.40).

P_14_^4−^ (symmetry *C_i_*) as found in [(Cp′′_2_La)_2_(*μ*_4_‐*ƞ* ^2^:*ƞ* ^2^:*ƞ* ^2^:*ƞ* ^2^P_14_)]^2−^ is formally composed of two P_7_^2−^ units connected to each other by a P─P single bond (SEN(P7─P7’): 1.03). The remaining four coordination sites of P1, P1‘, P3, and P3‘ serve as coordination centers to the two LaCp‘‘_2_ units. This finding is in line with their negative partial charges (Q(P1), Q(P3): −0.45). The shared electron number of the La─P bonds is determined to be 0.55 (SEN(La1─P1) and SEN(La1─P3)); these bonds are strengthened by 3‐center contributions (SEN(La1─P1─P2), SEN(La1─P3─P2): 0.12; SEN(La1─P1─P4), and SEN(La1─P3─P5): 0.18). The predominantly ionic character of the La─P bonds with a slight covalent component (Q(La1): +0.82) is well supported by the isosurface plots of the corresponding localized MOs (Figure 4). The P─P bonds are approximately of single bond character (SEN values between 1.03 and 1.21).

**Figure 4:**
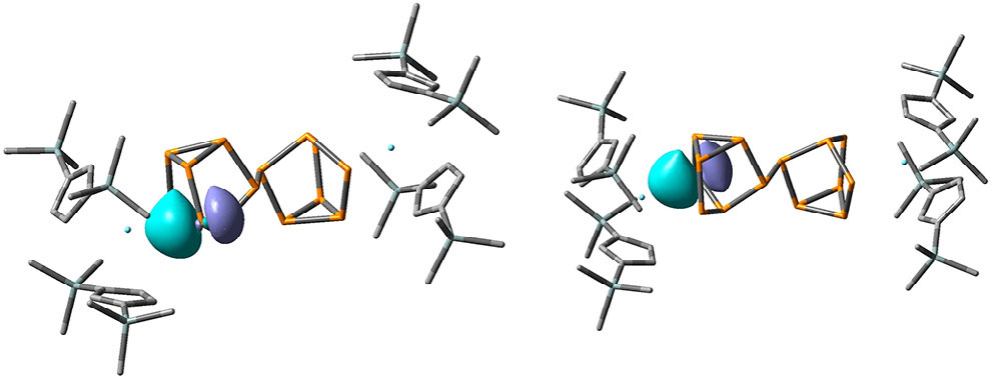
Isosurface plots (values ± 0.06 e) of the localized MOs (LMO 344 and 345) of **1** showing pronounced La1─P bonding.

In summary, we have demonstrated a novel synthetic strategy to obtain molecular organo‐lanthanide compounds coordinated by the large P_14_^4−^ polyphosphide Zintl anion. These compounds represent the largest organo‐f‐element polyphosphides known to date. The key steps within this strategy are the reactions of the three and four electron reducing agents of the early nonclassical divalent lanthanides with white phosphorus. The formation of a P_14_^4−^ Zintl anion is observed with both reducing agents. Only carefully tuned reaction conditions lead to the formation of these isolable and unprecedented products. This contributes to a better understanding of the formation and properties of polyphosphide compounds. While the reaction of the 1‐electron reducing agent [(*η* ^5^‐C_5_Me_5_)_2_Sm] with P_4_ leads to realgar‐type [{(*η* ^5^‐C_5_H_5_)_2_Sm}_4_P_8_],^[^ ^28^ ^]^ treatment of P_4_ with the multielectron reducing agents **I** and **II** resulted in the large P_14_^4−^ polyphosphide Zintl anion, clearly indicating the influence of the reducing agent on the product formation.

## Supporting Information

The authors have cited additional references within the Supporting Information.^[^ ^52^, ^53^, ^54^, ^55^, ^56^, ^57^, ^58^, ^59^, ^60^, ^61^ ^]^

## Conflict of Interests

The authors declare no conflict of interest.

## Supporting information



Supporting Information

Supporting Information

## Data Availability

Data for this paper are available at radar4chem [https://radar.products.fiz‐karlsruhe.de/] at https://doi.org/10.22000/hu6agwk1qnzxc1e7, reference number 1022000.
