# Supplementary material for: Synthesis of Molecular Organo‐f‐Element‐Polyphosphides with Nonclassical Divalent Lanthanide Precursors
Source: Angew Chem Int Ed Engl. 2025 May 19;64(29):e202503403. doi: 10.1002/anie.202503403 (PMC12258692; doi:10.1002/anie.202503403)
Supplement: Supplementary file 1 — Supporting Information [file ANIE-64-e202503403-s002.pdf]

Supporting Information

Synthesis of Molecular Organo-f-Element-Polyphosphides with Non-Classical Divalent Lanthanide Precursors

David Frick,[a] Elena Pross,[a] Ralf Köppe[a] and Peter W. Roesky* [a, b]

[a] D. Frick, E. Pross, Dr. R. Köppe, Prof. Dr. P.W. Roesky
Institute of Inorganic Chemistry
Karlsruhe Institute of Technology (KIT)
Kaiserstr. 12, 76131 Karlsruhe, Germany
E-mail: roesky@kit.edu

[b] Prof. Dr. P. W. Roesky
Institute of Nanotechnology (INT)
Karlsruhe Institute of Technology (KIT)
Kaiserstr. 12, 76131 Karlsruhe, Germany

1. Table of contents

1. Table of contents S1

2. General considerations S2

3. Synthesis of [K(18 Crown 6)]$_2$[(Cp''$_2$La)$_2$(μ_4-η^2:η^2:η^2:η^2-P$_{14}$)] (1) and [K(18 Crown 6)][(Cp''$_2$La)$_2$(μ-η^3:η^3-P$_3$)](3) S3

3.1 Analytics of [K(18 Crown 6)]$_2$[(Cp''$_2$La)$_2$(μ_4-η^2:η^2:η^2:η^2-P$_{14}$)] (1) S3

3.2 Analytics of [K(18 Crown 6)][(Cp''$_2$La)$_2$(μ-η^3:η^3-P$_3$)] (3) S4

4. Synthesis of [K(18 Crown 6)]$_2$[(Cp''$_2$Ce)$_2$(μ_4-η^2:η^2:η^2:η^2-P$_{14}$)] (2) and [K(18 Crown 6)][(Cp''$_2$Ce)$_2$(μ-η^3:η^3-P$_3$)] (4) S4

4.1. Analytics of [K(18 Crown 6)]$_2$[(Cp''$_2$Ce)$_2$(μ_4-η^2:η^2:η^2:η^2-P$_{14}$)] (2) S4

4.2. Analytics of [K(18 Crown 6)][(Cp''$_2$Ce)$_2$(μ-η^3:η^3-P$_3$)] (4) S5

5. NMR Spectra S6

6. IR Spectra S12

7. X-ray Crystallographic Studies S14

8. Quantum chemical Calculations S17

9. References S24

2. General considerations

All manipulations of water- and air-sensitive compounds were performed with the exclusion of moisture and oxygen in a dry Argon atmosphere. For this purpose, a dual-manifold Schlenk line interfaced to a high vacuum (10^{-3} mbar) oil pump or an argon-filled MBraun glove box were used.

Hydrocarbon solvents were dried by using an MBraun solvent purification system (SPS 800), and stored over Molecular sieves (0.4 nm, rod shaped). Tetrahydrofuran was distilled under nitrogen atmosphere from potassium benzophenoneketyl before the atmosphere was replaced with the freeze pump thaw method to argon and stored over Molecular sieves (0.4 nm, rod shaped).

A Bruker Tensor 37 FTIR spectrometer equipped with a room temperature DLaTGS detector and a diamond attenuated total reflection (ATR) unit was used to record infrared (IR) spectra in the 3600-400 cm^{-1} region.

NMR spectra were recorded on Bruker spectrometers (Avance III 300 MHz, Avance III 400 MHz). Chemical shifts are referenced to residual protio solvent (^{1}H) or solvent (^{13}C) signals. They are reported relative to tetramethylsilane (^{1}H, ^{13}C, ^{29}Si). ^{31}P signals are reported relative to 85 % H_3PO_4. Unless otherwise stated, all NMR spectra were measured at 298K. Signal multiplicity is indicated as s = singlet, d = doublet, t = triplet, m = multiplet and br = broad. The assignments have been determined on the basis of unambiguous chemical shifts and coupling patterns.

Raman spectra were recorded in the region of 4000-20 cm^{-1} on a Bruker MultiRam spectrometer equipped with a Nd:YAG laser (λ = 1064 nm) and a germanium detector at a resolution of 2 cm^{-1} . The powdered crystalline sample materials were flame sealed in a glass tube. The laser energy was adjusted to values between 20 and 200 mW depending on the FID amplitude and laser focusing.

Elemental analysis were carried out with an Elementar Unicube. The compounds were prepared using tin boats in argon-filled glove boxes.

The starting materials [K(18-Crown-6)][(Cp“$_2$La)$_2$(μ-η^6:η^6-C_6H_6)][1], [K(18-Crown-6)]$_2$[(Cp“$_2$Ce)$_2$(μ-η^6:η^6-C_6H_6)][2-3] were prepared using literature procedures.

3. Synthesis of [K(18 Crown 6)]$_2$[(Cp''$_2$La)$_2$(μ$_4$-η^2:η^2:η^2:η^2-P$_{14}$)] (1) and [K(18 Crown 6)][(Cp''$_2$La)$_2$(μ-η^3:η^3-P$_3$)](3)

THF was introduced to a mixture of [K(18-Crown-6)][(Cp''$_2$La)$_2$(μ-η^6:η^6-C_6H_6)] (200.0 mg, 0.133 mmol, 1.00 equiv.) and freshly sublimed P_4 (24.8 mg, 0.200 mmol, 1.50 equiv). The resulting reaction solution was stirred for 48 h at RT. All volatiles were removed under reduced pressure and the remaining solid was washed with 10 ml of *n*-heptane. Then the product **1** was extracted by washing 3 times with 5 ml of toluene. The product **3** was extracted out of the remaining solid after the extraction of **1** with 2 times 5 ml of 1,2-Difluorobenzene. Orange crystals of **1** and red crystals of **3** were obtained by slow evaporation of the extraction solutions at ambient temperatures.

Yield of 1: 91 mg (31.6 %).

Yield of 3: 46 mg (22.7 %)

3.1 Analytics of [K(18 Crown 6)]$_2$[(Cp''$_2$La)$_2$(μ$_4$-η^2:η^2:η^2:η^2-P$_{14}$)] (1)

IR(ATR): 2945 (m), 2913 (m), 2858 (m), 1452 (vw), 1351 (w), 1245 (w), 1106 (vs), 961 (w), 911 (w), 828 (m), 746 (w), 462 (w).

Raman: Due to Raman fluorescence no meaningful Raman spectra were obtained.

^{1}H-NMR (400 MHz, THF-d_8) δ (ppm): 6.11-6.09 (m, 4H, C*H*Cp), 6.03-6.00 (m, 8H, C*H*Cp), 3.58 (s, 48H, CH_2), 0.08 (s, 72H, CH_3).

^{13}C-NMR (100 MHz, THF-d_8) δ (ppm): 137.0 (s, *C*Si), 131.4 (s, *C*Si), 121.0 (s, *C*HCp), 116 (s, *C*HCp), 115.3(s, *C*HCp), 71.4 (s, CH_2), 0.9 (s, SiCH_3).

^{31}P-NMR (162MHz, THF-d_8) δ (ppm): 467.7 (s), 63.08 – 57.7 (m), 40.8 – 39.4 (m), 5.9 (s), -23.4 (s), -25.5 (s), -34.3 – -37.2 (m), -49.2 (s), -56.9 – -57.8 (m), -63.0 (s), -69 – -70.9 (m), -122.5 (s), -134.6 – -136.8 (m), -146.6 (s), -151.9 (s), -174.1 – -175.9 (m), -237.7 (s), -238.3 (s).

^{31}P-NMR (162 MHz, THF-d_8, 193K) δ (ppm): 467.2 (s), 104.6 (d, J = 354.9 Hz), 68.3 (d, J = 339.6 Hz), 57.8 (d, J = 685.4 Hz), 46.3 (d, J = 449.9 Hz), 35.1 (t, J = 246.0 Hz), 11.4 – -1.6 (m), -3.4 (s), -5.4 (d, J = 41.7 Hz), -7.5 (s), -16.3 (s), -34.5 (s), -36.6 – -42.6 (m), -62.5 (d, J = 767.3 Hz), -69.8 (s), -72.1 (dd, J = 1080.3, 51.9 Hz), -74.5 (s), -76.0 (s), -78.4 (s), -80.5 (s), -108.2 (d, J = 72.3 Hz), -109.5 (d, J = 73.6 Hz), -110.8 (d, J = 67.2 Hz), -111.6 – -112.5 (m), -112.7 – -113.3 (m), -114.2 (s), -118.8 (s), -121.1 (s), -123.1 (d, J = 17.5 Hz), -137.1 (s), -139.2 (d, J = 29.7 Hz), -141.4 (s), -142.7 (s), -147 – -153.0 (m), -157.2 (s), -159.4 (d, J = 66.5 Hz), -161.6 (s), -171.8 – -179.1 (m), -180.0 (s), -194.5 – -209.5 (m), -241.8 (s), -260.3(s).

^{29}Si-NMR (80 MHz, THF-d_8) δ (ppm): -14.6 (s, *Si*CH_3).

Elementary Analysis: Due to carbon values that were consistently too low, no usable data out of elemental analysis could be obtained.

3.2 Analytics of [K(18 Crown 6)][(Cp“$_2$La)$_2$(μ-η^3:η^3-P$_3$)] (3)

IR(ATR): 2947 (vw), 2891 (vw), 2367 (vw), 2280 (vw), 1640 (vw), 1444 (vw),1348 (vw), 1303 (m), 1234 (vs), 1145 (s), 1105 (s), 984 (m), 923 (w), 824 (m), 746 (w), 684 (vw), 630 (vw), 514 (vw), 470 (vw).

Raman: Due to Raman fluorescence no meaningful Raman spectra were obtained.

^{1}H-NMR (400 MHz, THF-d$_8$) δ (ppm): 6.69-6.64 (4H, C*H*Cp), 6.21-6.13 (8H, C*H*Cp), 3.61 (24H, C*H*$_2$), 0.25 (72H, C*H*$_3$).

^{13}C-NMR (100 MHz, THF-d$_8$) δ (ppm): 129.4 (*C*Si), 121.4 (*C*HCp), 120.9 (*C*HCp), 71.4 (CH$_2$), 2.2 (SiCH$_3$).

^{31}P-NMR (162 MHz, THF-d$_8$) δ (ppm): -156.39 (s).

Elementary Analysis: Due to carbon values that were consistently too low, no usable data out of elemental analysis could be obtained.

4. Synthesis of [K(18 Crown 6)]$_2$[(Cp“$_2$Ce)$_2$(μ$_4$-η^2:η^2:η^2:η^2-P$_{14}$)] (2) and [K(18 Crown 6)][(Cp“$_2$Ce)$_2$(μ-η^3:η^3-P$_3$)] (4)

THF was introduced to a mixture of [K(18-Crown-6)$_2$][(Cp“$_2$Ce)$_2$(*μ*-*η*^{6}:*η*^{6}-C$_6$H$_6$)] (200.0 mg, 0.111 mmol, 1.00 equiv.) and freshly sublimed P$_4$ (27.5 mg, 0.222 mmol, 2.00 equiv). The resulting reaction solution was stirred for 48 h at RT. All volatiles were removed under reduced pressure and the remaining solid was washed with 10 ml of n-heptane. Then the product **2** was extracted 3 times with 5 ml of toluene. The product **4** was extracted out of the remaining solid after the extraction of **2** with 2 times 5 ml of 1,2-Difluorobenzene. Orange crystals of **2** and red crystals of **4** were obtained by slow evaporation of the extraction solutions at ambient temperatures.

Yield of 2: 96 mg (40.1 %)

Yield 4: 20 mg (11.91 %)

4.1. Analytics of [K(18 Crown 6)]$_2$[(Cp“$_2$Ce)$_2$(μ$_4$-η^2:η^2:η^2:η^2-P$_{14}$)] (2)

IR(ATR): 2948 (m), 2920 (s), 2853 (s), 1466 (vw), 1452 (vw), 1436 (vw), 1402 (vw), 1350 (w), 1243 (m), 1104 (vs), 1077 (m), 1039 (vw), 999 (vw), 960 (m), 921 (w), 825 (vs), 749 (m), 686 (vw), 637 (vw), 473 (vw).

Raman: Due to Raman fluorescence no meaningful Raman spectra were obtained.

NMR: Due to the paramagnetic behavior of Ce(III) atoms, no meaningful data could be obtained.

Elementary Analysis: Due to carbon values that were consistently too low, no usable data out of elemental analysis could be obtained.

4.2. Analytics of [K(18 Crown 6)][(Cp''$_2$Ce)$_2$(μ-η^3:η^3-P$_3$)] (4)

IR(ATR): 3851 (vw), 3743 (vw), 3661 (vw), 2952 (s), 2892 (s), 2362 (vw), 2331 (vw), 1458 (w), 1446 (w), 1392 (vw), 1351 (w), 1243 (s), 1104 (vs), 992 (vw), 956 (m), 921 (w), 824 (vs), 748 (m), 684 (w), 629 (w), 469 (vw).

Raman: Due to Raman fluorescence no meaningful Raman spectra were obtained.

NMR: Due to the paramagnetic behavior of Ce(III) atoms, no meaningful data could be obtained.

Elementary Analysis: Due to carbon values that were consistently too low, no usable data out of elemental analysis could be obtained.

5. NMR Spectra

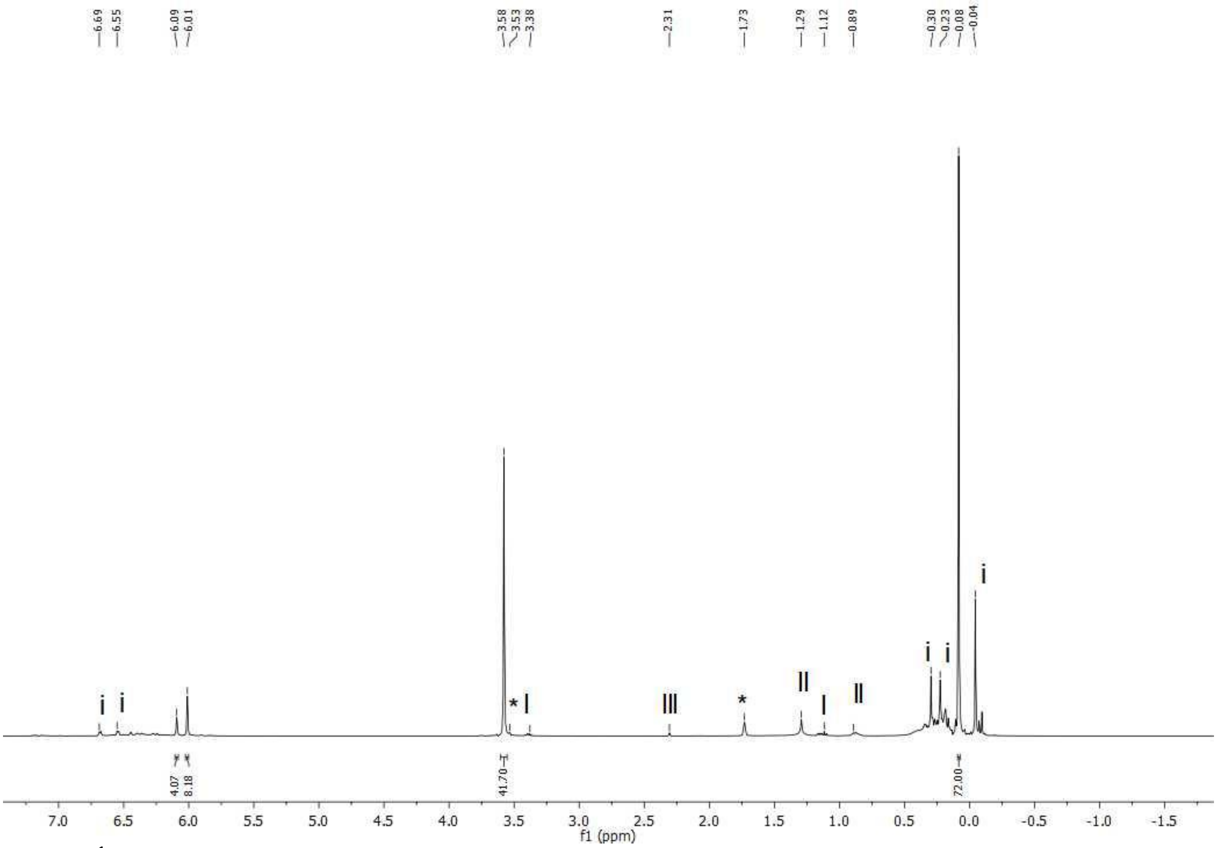

Fig. S1: ^{1}H-NMR-spectrum of **1** THF-d_8. (* solvent signal; I remaining Et_2O signal from extraction; II remaining heptane signal from washing step; III remaining toluene signal from extraction; i signal from unknown decomposition product).

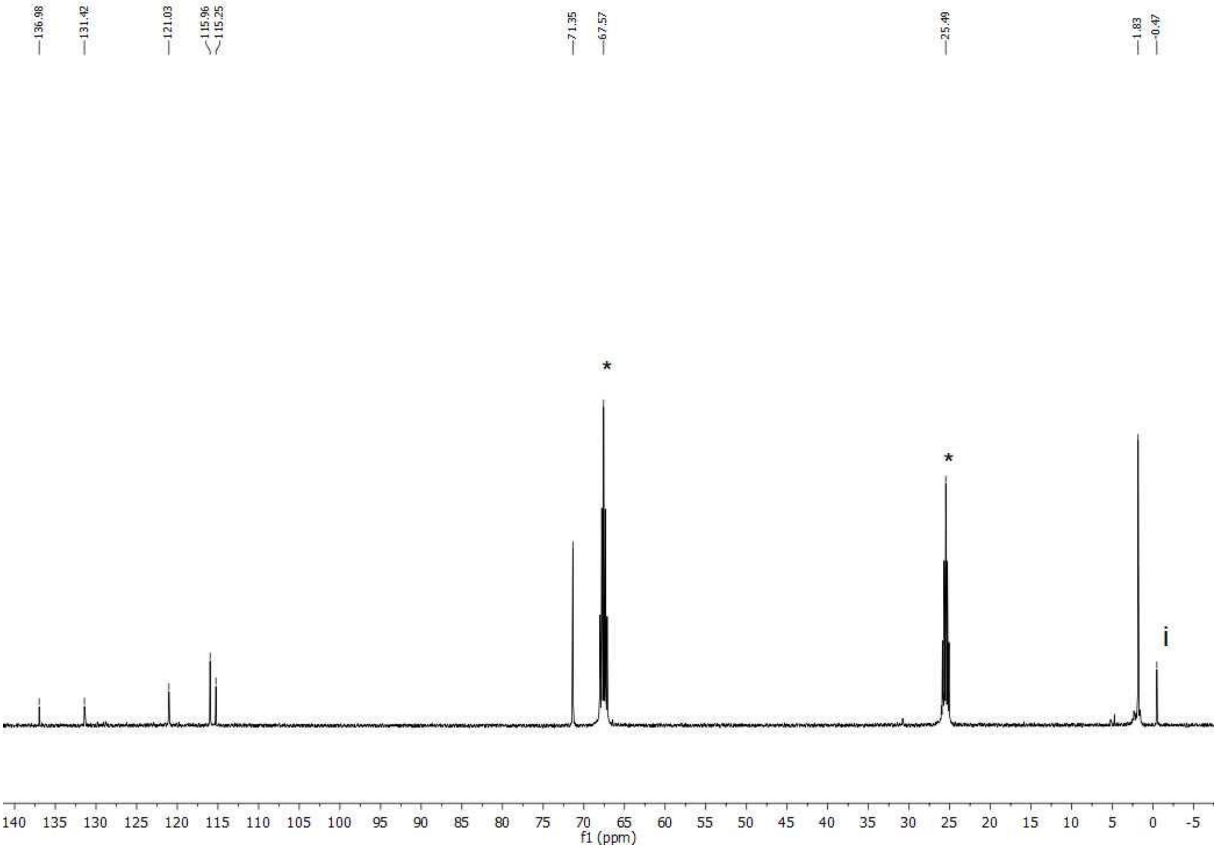

Fig. S2: $^{13}C\{^{1}H\}$-NMR-spectrum of **1** in THF-d_8. (* solvent signal; i signal of unknown decomposition product).

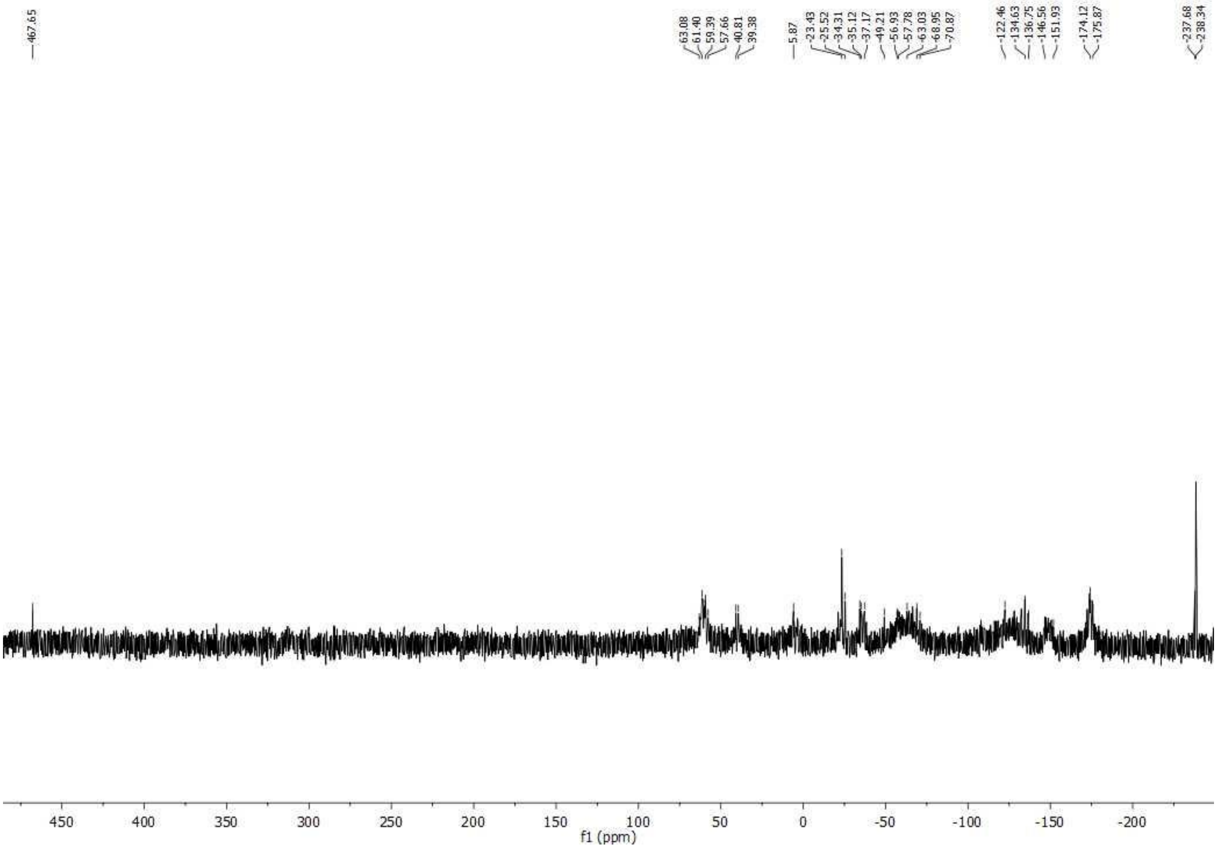

Fig. S3: $^{31}P\{^{1}H\}$-NMR-spectrum of **1** in THF-d_8.

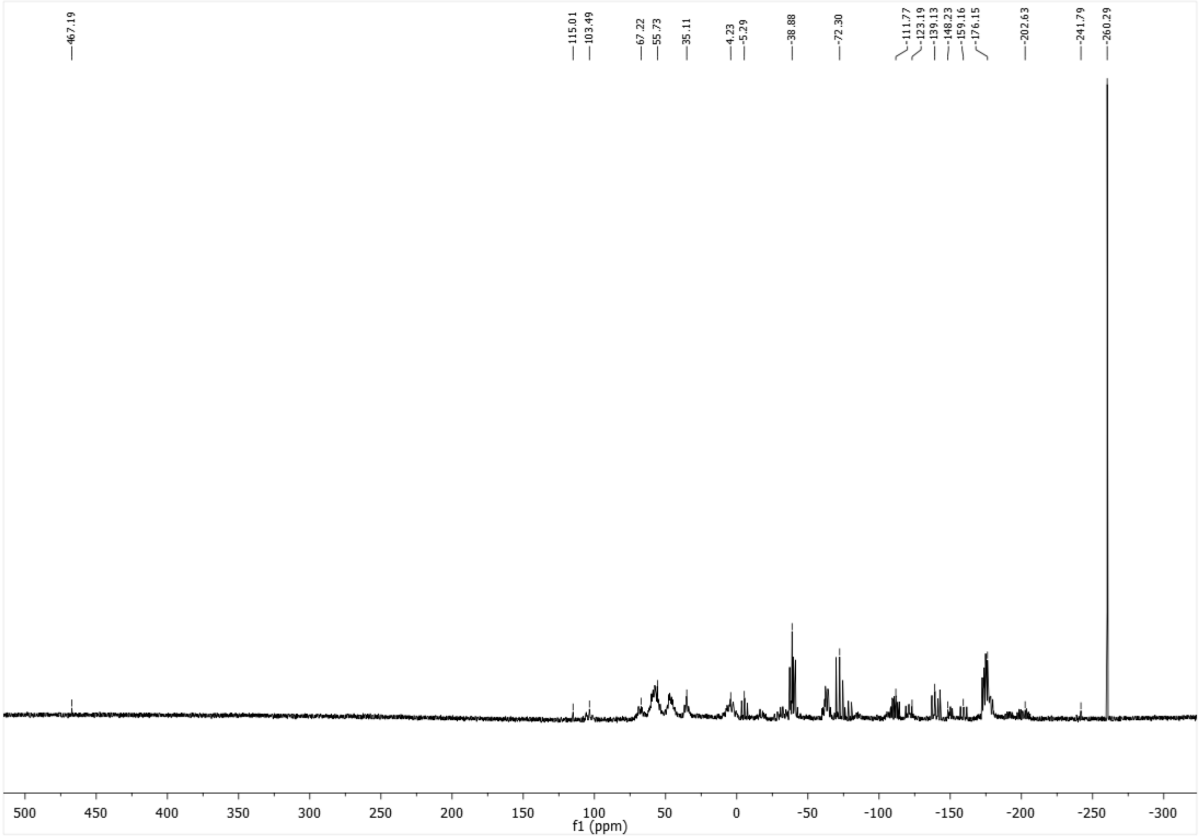

Fig. S4: $^{31}P\{^1H\}$-NMR-spectrum of **1** in THF-d_8 at 193K.

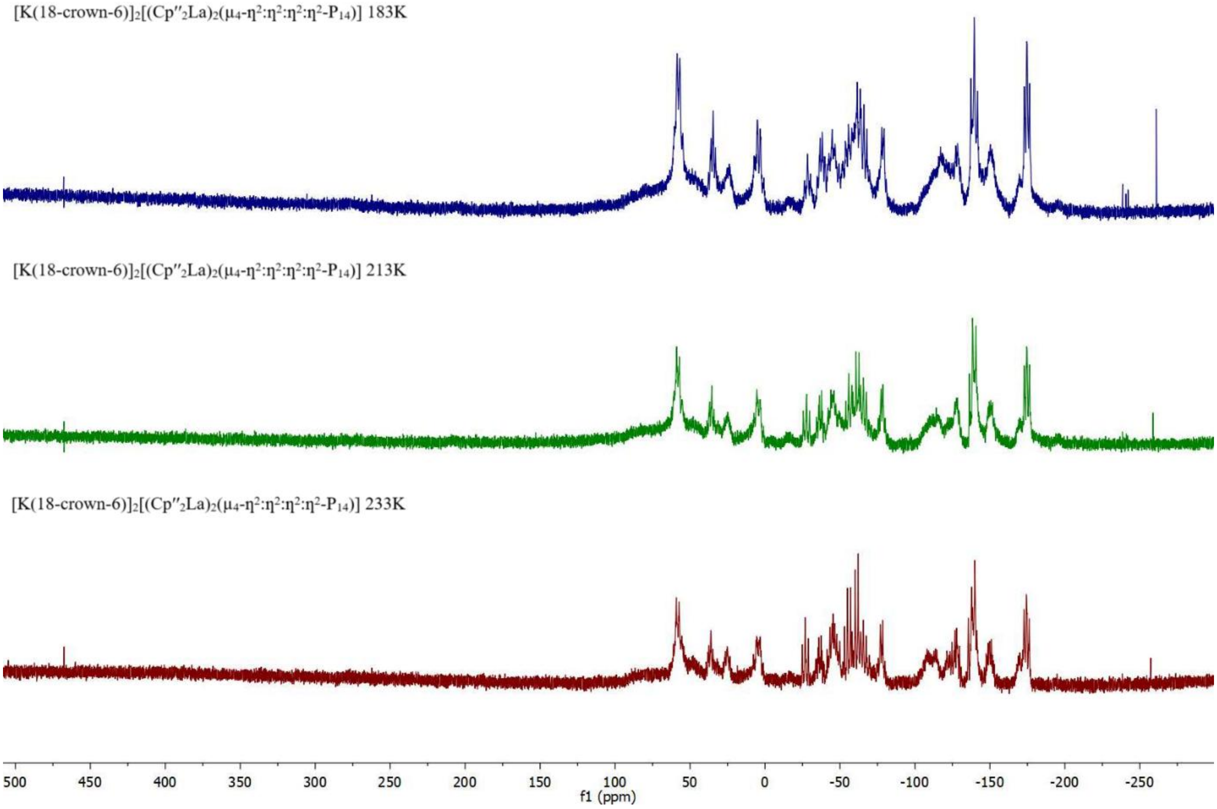

Fig. S5: $^{31}P\{^1H\}$-NMR-spectra of **1** in THF-d_8 at 233K, 213K and 183K.

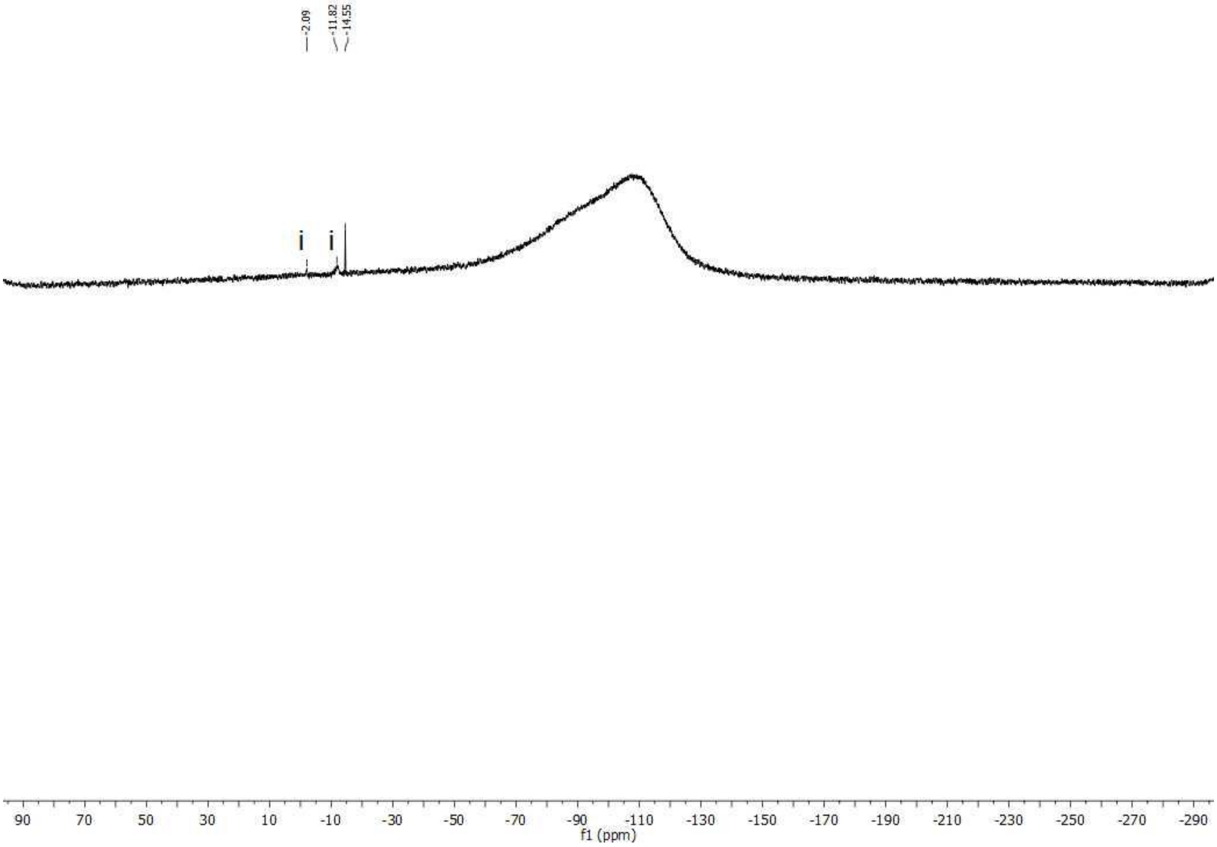

Fig. S6: $^{29}Si\{^1H\}$-NMR-spectrum of **1** THF-d_8. (i signals of unknown decomposition products.)

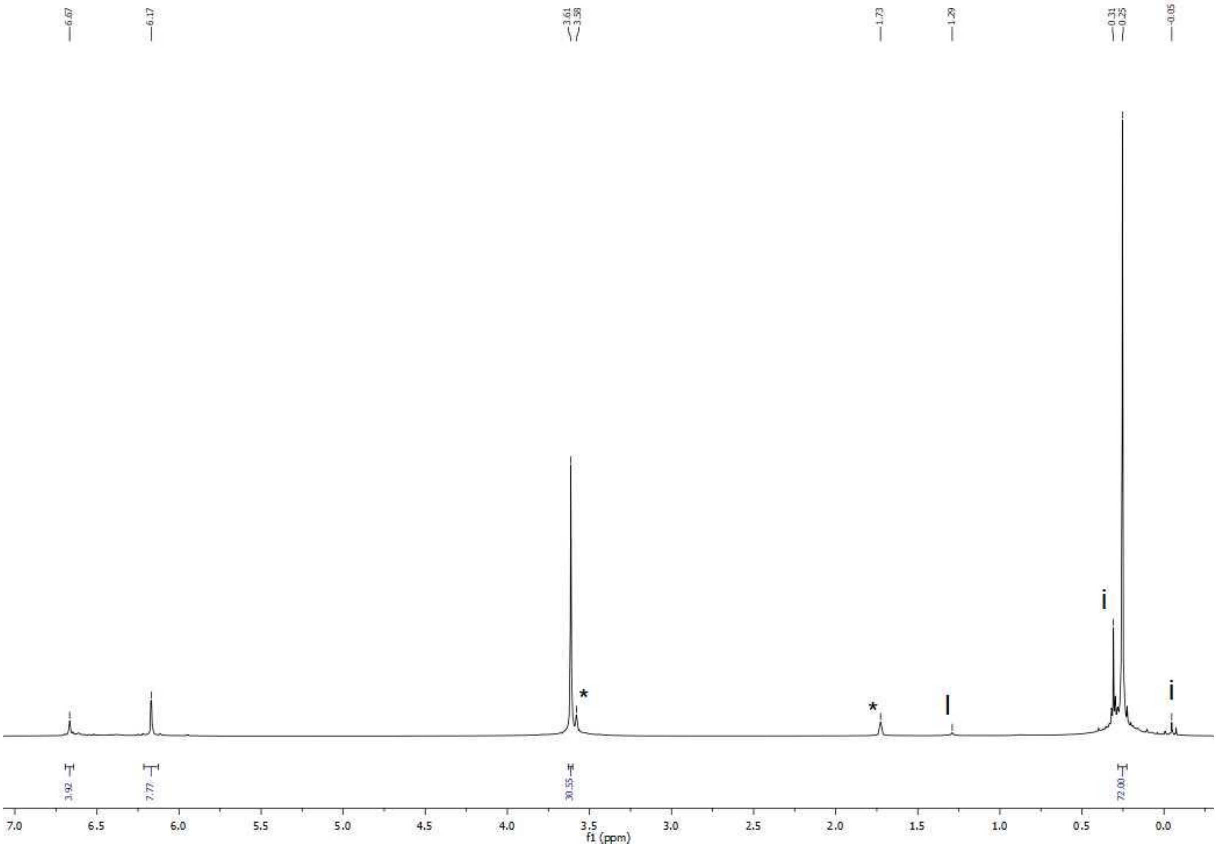

Fig. S7: ^{1}H-NMR-spectrum of **3** THF-d$_8$. (* solvent signal; I remaining heptane signal from washing step; i signal from unknown decomposition product).

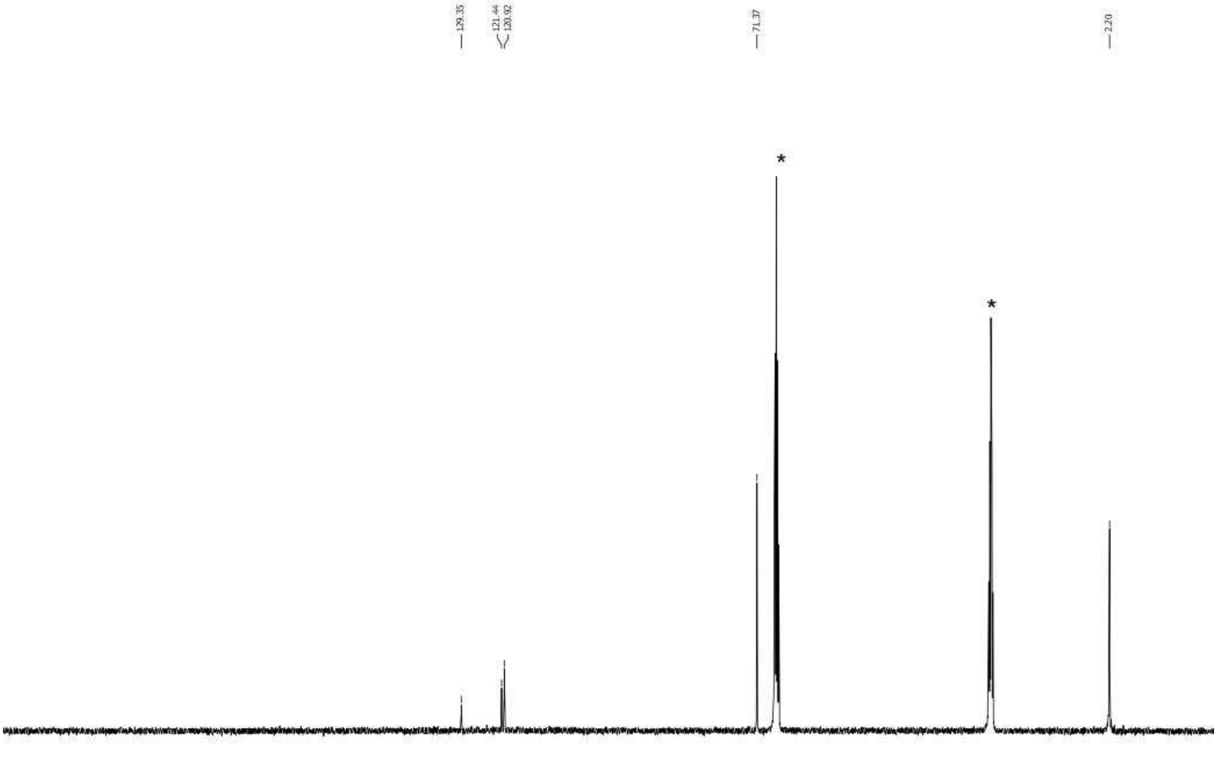

Fig. S8: ^{13}C{^{1}H}-NMR-spectrum of **3** in THF-d$_8$. (* solvent signal).

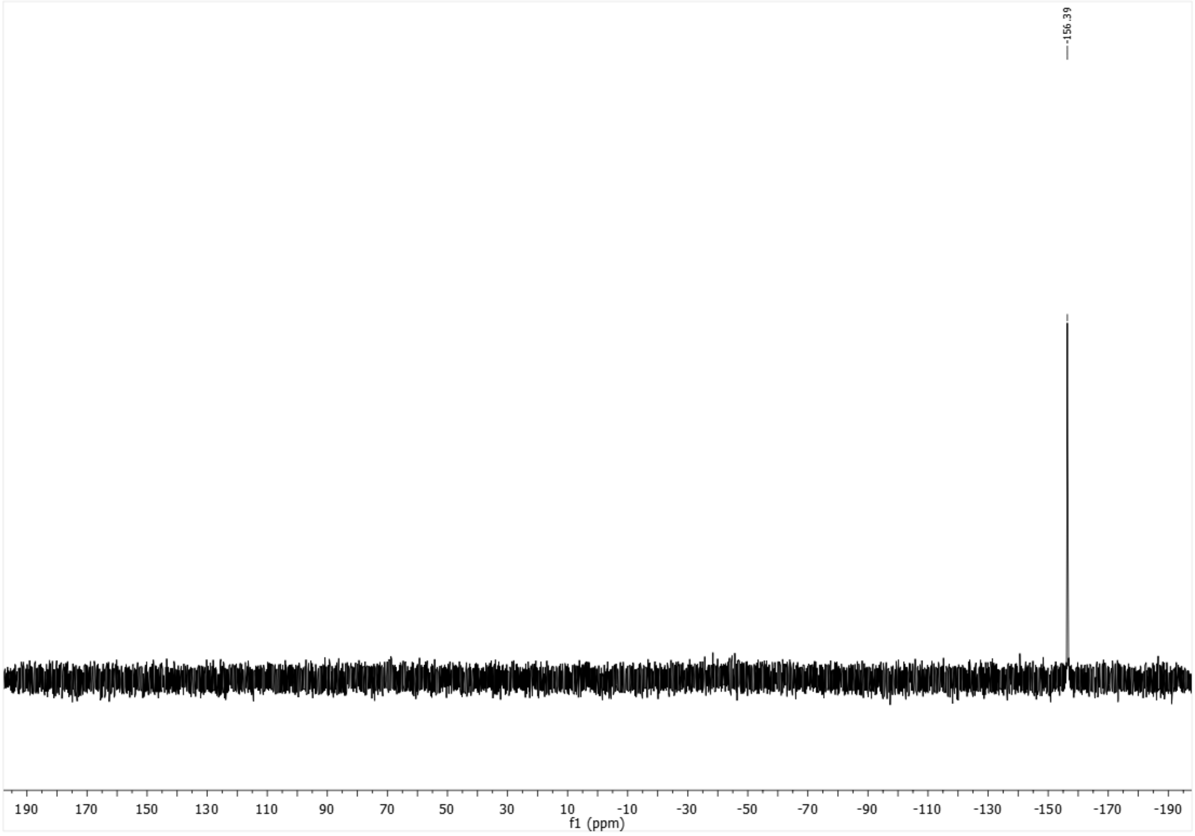

Fig. S9: $^{31}P\{^{1}H\}$-NMR-spectrum of **3** in THF-d_8.

6. IR Spectra

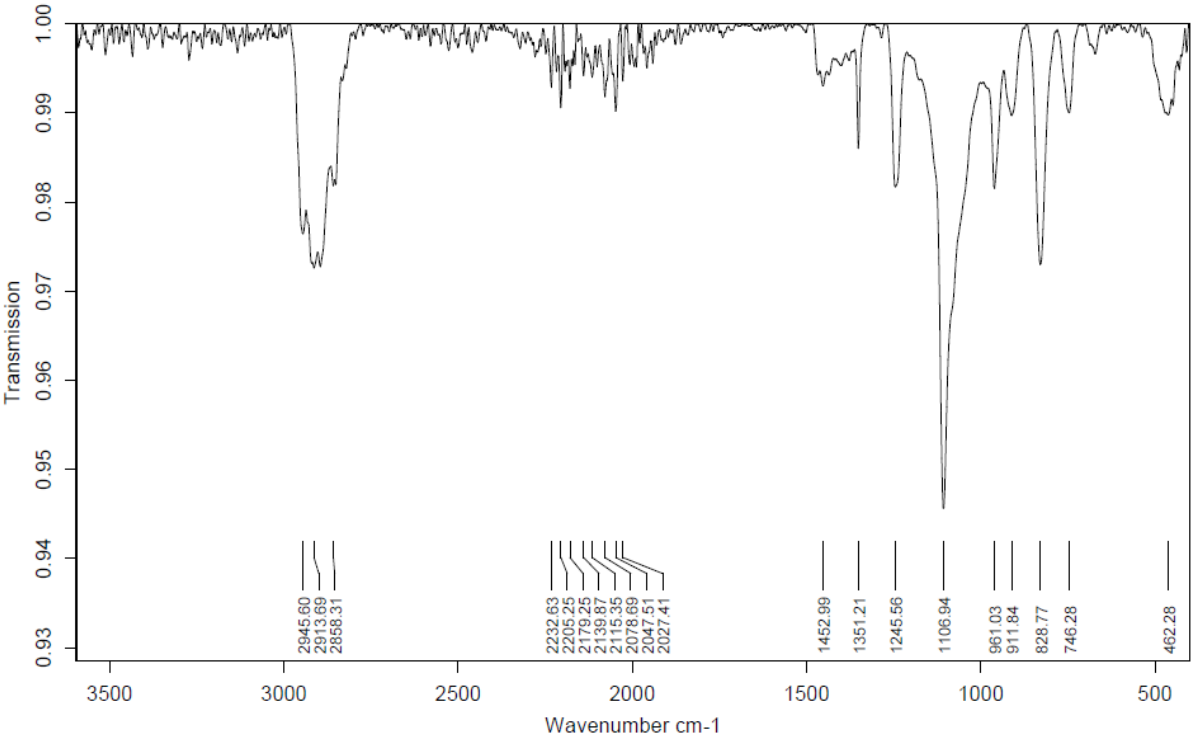

Fig. S10: IR-Spectrum (ATR) of **1**

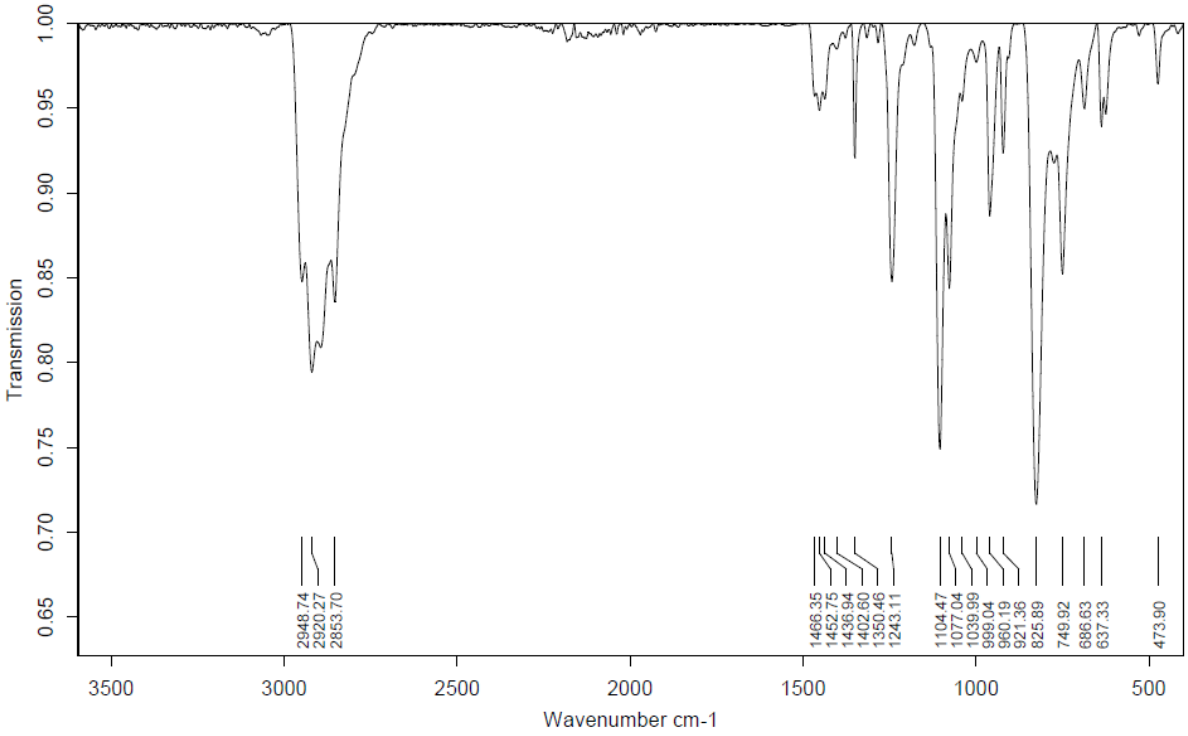

Fig. S11: IR-Spectrum (ATR) of **2**

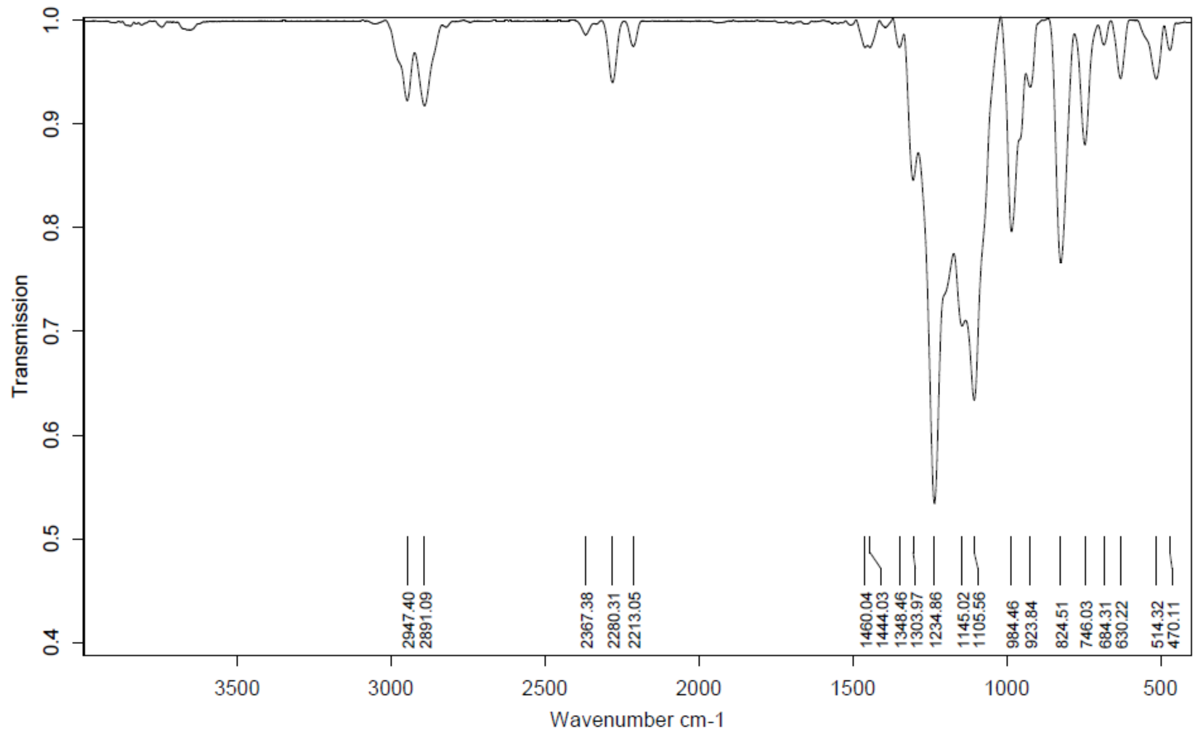

Fig. S12: IR-Spectrum (ATR) of **3**

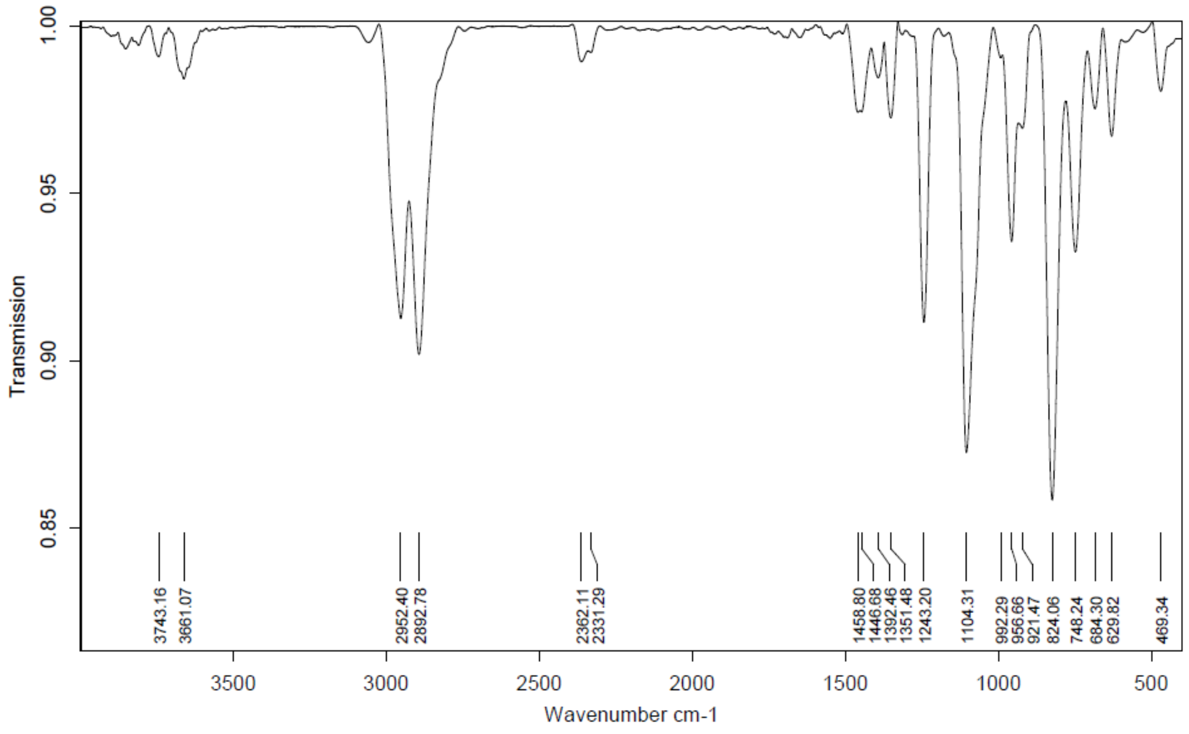

Fig. S13: IR-Spectrum (ATR) of **4**

7. X-ray Crystallographic Studies

Suitable crystals were selected under an optic microscope equipped with polarizing filters, covered in mineral oil (Aldrich) and mounted on a MiTeGen holder. The crystals were directly transferred into the cold stream of a STOE StadiVari (100 K or 111 K) diffractometer. The X-ray source is a Mo-sealed tube MoGenix 3D. All structures were solved using the programs SHELXS/T and Olex2 1.5.[4-6] The remaining non-hydrogen atoms were located from successive difference Fourier map calculations. SHELXL or olex2.refine were the programs used to carry out the refinements by using full-matrix least-squares techniques on F2 and the Levenberg-Marquard method. In all structures the locations of the largest peaks in the final Fourier map calculations, as well as the magnitude of the residual electron densities, were of no chemical significance.

Crystallographic data for the structures reported in this paper have been deposited with the Cambridge Crystallographic Data Centre as a supplementary publication no. 2422293-2422296. Copies of the data can be obtained free of charge on application to CCDC, 12 Union Road, Cambridge CB21EZ, UK (fax: (+(44)1223-336-033; email: deposit@ccdc.cam.ac.uk).

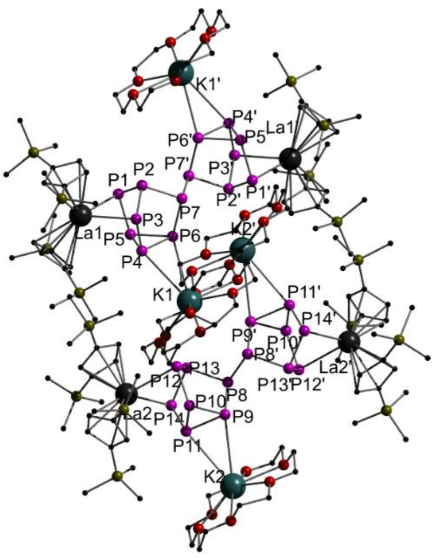

Fig. S14: Molecular structure of **1** in the solid state. Hydrogen atoms and solvent molecules are omitted for clarity. Disorder of [K(18-crown-6)]$^+$ over two parts with an occupancies of 0.8:0.2. Selected bond lengths [Å] and angles [°]: La1-P1 3.0615(7), La1-P3 3.0192(7), K1-P4 3.6694(10), K1-P6 3.6892(11), La2-P12 3.0020(7), La2-P14 3.0602(7), K2-P9 3.4080(9), K2-P11 3.7902(9), P4-P6 2.2238(9), P4-P5 2.2541(10), P4-P3 2.1807(9), P5-P6 2.2333(9), P7-P6 2.1946(9), P5-P1 2.1557(10), P2-P1 2.1775(9), P2-P3 2.1875(9), P2-P7 2.2073(9), P7-P7´ 2.2343(13), P1-La1-P3 69.77(2), P4-K1-P6 35.18(2), P9-P10 2.2305(10), P9-P11 2.2334(10), P10-P14 2.1588(10), P14-P13 2.1794(9), P13-P12 2.1903(9), P8-P13 2.1994(9), P8-P9 2.1961(10), P11-P12 2.1768(10), P8-P8´ 2.2299(13), P14-La2-P12 70.19(2), P9-K2-P11 35.65(2).

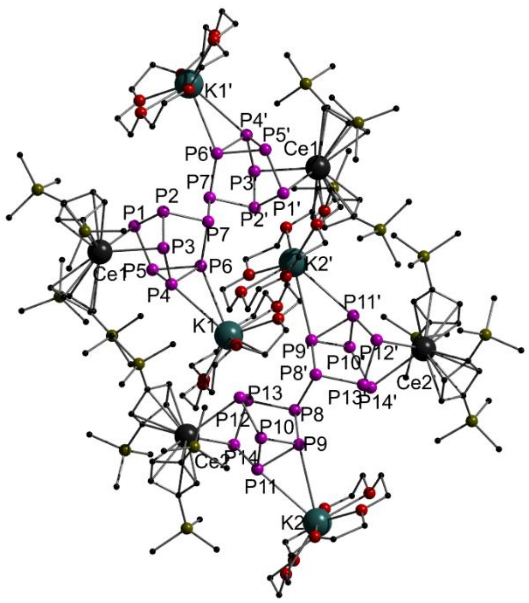

Fig. S15: Molecular structure of **2** in the solid state. Hydrogen atoms and solvent molecules are omitted for clarity. Disorder of [K(18-crown-6)]$^+$ over two parts with an occupancies of 0.5:0.5. Selected bond lengths [Å] and angles [°]: Ce1-P1 2.9773(13), Ce1-P3 3.0353(12), K1-P4 3.781(2), K1-P6 3.4018(15), Ce2-P12 3.0431(13), Ce2-P14 2.9938(12), K2-P9 3.6500(2), K2-P11 3.638(2), P4-P6 2.233(2), P4-P5 2.243(2), P3-P5 2.159(2), P4-P3 2.159(2), P5-P6 2.233(2), P7-P6 2.194(2), P4-P1 2.174(2), P2-P1 2.190(2), P2-P3 2.179(2), P2-P7 2.200(2), P7-P7´ 2.228(2), P1-Ce1-P3 70.53(3), P4-K1-P6 35.73(3), P9-P11 2.227(2), P9-P10 2.233(2), P10-P11 2.250(2), P10-P12 2.153(2), P11-P14 2.183(2), P9-P8 2.191(2), P8-P13 2.207(2), P12-P13 2.174(2), P13-P14 2.187(2), P8-P8´ 2.233(2), P1-Ce1-P3 70.54(3), P6-K1-P4 35.73(3), P12-Ce2-P14 70.04(3), P9-K2-P11 35.59(3).

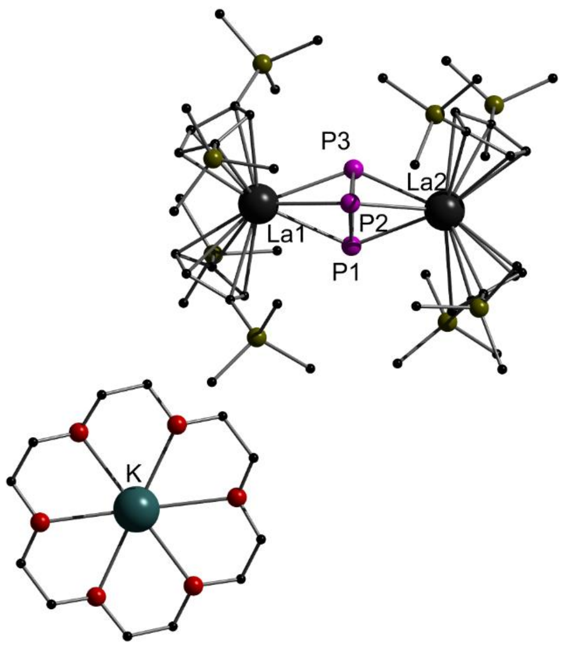

Fig. S16: Molecular structure of **3** in the solid state. Hydrogen atoms and solvent molecules are omitted for clarity. Selected bond lengths [Å] and angles [°]: La1-P1 2.9157(6), La1-P2 2.9443(6), La2-P1 2.9007(6), La2-P2 2.9317(6), P1-P2 2.1887(9), P1-P3 2.1907(8), P2-P3 2.1833(8), P1-La1-P2 43.86(2), P1-La2-P2 44.08(2), P2-P1-P3 59.81(3), P3-P2-P1 60.14(3), P2-P3-P1 60.05(3), La1-P1-La2 131.33(2), La1-P2-La2 128.82(2), La1-P3-La2 126.87(2), La1-Ct(Cp´´) 2.60328(8), La1-Ct(Cp´´) 2.59903(9), Ct(Cp´´)-La1-Ct(Cp´´) 118.335(5), La2-Ct(Cp´´) 2.59708(8), La2-Ct(Cp´´) 2.61302(8), Ct(Cp´´)-La2-Ct(Cp´´) 119.219(5).

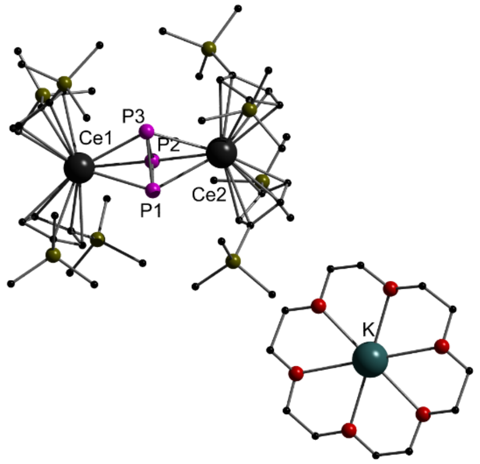

Fig. S17: Molecular structure of **4** in the solid state. Hydrogen atoms and solvent molecules are omitted for clarity. Selected bond lengths [Å] and angles [°]: Ce1-P1 2.8913(7), Ce1-P2 2.9190(7), Ce2-P1 2.8745(7), Ce2-P2 2.9066(7), P1-P2 2.1871(11), P1-P3 2.1888(10), P2-P3 2.1782(10), P1-Ce1-P2 44.22(2), P1-Ce2-P2 44.45(2), P2-P1-P3 59.71(4), P3-P2-P1 60.19(3), P2-P3-P1 60.11(4), Ce1-P1-Ce2 130.88(3), Ce1-P2-Ce2 128.37(2), Ce1-P3-Ce2 126.53(2), Ce1-Ct(Cp´´) 2.57405(9), Ce1-Ct(Cp´´) 2.57448(9), Ct(Cp´´)-Ce1-Ct(Cp´´) 118.221(5), Ce2-Ct(Cp´´) 2.57201(9), Ce2-Ct(Cp´´) 2.58067(9), Ct(Cp´´)-Ce2-Ct(Cp´´) 119.092(5).

Table S1: Crystal data and structure refinement of **1**,**2**,**3** and **4**.

**Compound**	**1**	**2**	**3**	**4**
**Formula**	$C_{143}H_{280}K_4La_4O_{24}P_{28}Si_{16}$	$C_{143}H_{280}Ce_4K_4O_{24}P_{28}Si_{16}$	$C_{80}H_{124}F_8KLa_2O_6P_3Si_8$	$C_{80}H_{124}Ce_2F_8KO_6P_3Si_8$
$D_{calc.}$**/g cm**$^{-3}$	1.374	1.384	1.313	1.322
μ**/mm**$^{-1}$	1.216	1.276	1.091	1.154
**Formula Weight**	4412.450	4417.13	1968.33	1970.75
**Colour**	orange	red	orange	red
**Shape**	plate	fragment	stick	plate
**Size/ mm**^{3}	0.362×0.167×0.025	0.17×0.098×0.03	0.282×0.147×0.069	0.164×0.091×0.037
T**/K**	100	111	100	100
**Crystal System**	triclinic	triclinic	monoclinic	monoclinic
**Space Group**	P-1	P-1	$P2_1/c$	$P2_1/c$
a**/Å**	11.2083(2)	11.1967(6)	13.1999(3)	13.1313(2)
b**/Å**	22.7802(5)	22.7632(17)	21.6993(7)	21.7316(6)
c**/Å**	23.8671(5)	23.7354(11)	34.7695(6)	34.6967(7)
α**/°**	115.010(2)	115.010(5)	90	90
β**/°**	97.984(2)	98.051(4)	91.2220(10)	91.098(2)
γ**/°**	98.124(2)	97.714(5)	90	90
V**/Å**^{3}	5331.4(2)	5300.6(6)	9956.7(4)	9899.4(4)
**Z**	1	1	4	4
**Wavelength/ Å**	0.71073	0.71073	0.71073	0.71073
**Radiation type**	Mo Kα	Mo Kα	Mo Kα	Mo Kα
**Θmin/°**	2.90	1.881	1.806	3.242
**Θmax/°**	30.13	30.266	28.195	27.827
**Measured Refl's.**	73222	59119	59194	60499
**Indep't Refl's**	26433	25483	22167	21581
R_{int}	0.0272	0.0508	0.0192	0.0297
**Parameters**	1069	1023	1003	1003
**Restrains**	18	0	15	0
**Largest Peak**	1.84	1.47	0.70	1.38
**Deepest Hole**	-1.19	-1.41	-0.66	-0.55
**GooF**	1.031	0.993	1.040	0.888
wR_2**(all data)**	0.0815	0.1188	0.0592	0.0598
wR_2	0.0744	0.1019	0.0562	0.0565
R_1**(all data)**	0.0517	0.1042	0.0425	0.0549
R_1	0.0333	0.0493	0.0259	0.0295

8. Quantum chemical Calculations

Quantum chemical RI-DFT calculations on $[(Cp''_2La)_2(\mu_4\text{-}\eta^2:\eta^2:\eta^2:\eta^2\text{-}P_{14})]^{2-}$ as well as P_7^{3-} were performed by means of the program system TURBOMOLE[7-8] using the RI-BP86

functional[9-11] including Grimme D3 dispersion correction[12]. The basis sets were of def2-TZVP quality for each atom as taken from the TURBOMOLE basis-set library. For Lanthanum an ECP of 46 electrons has been taken. Partial charges (Q) and shared electron numbers (SEN) were obtained using a population analysis based on occupation numbers[13] (choosing 1 modified atomic orbital (MAO) for H, 5 MAOs for C, 9 MAOs for Si and P, 8 MAOs for La). Localized molecular orbitals were obtained using the method of Pipek and Mezey[14].

Cartesian Coordinates of $[(Cp''_2La)_2(\mu_4\text{-}\eta^2:\eta^2:\eta^2:\eta^2\text{-}P_{14})]^{2-}$ (given in atomic units)

1.00695003581629	-8.89273070089235	36.57988047524839	la
2.14687198228873	-6.28122203289976	41.44020609700144	p
0.30637259346459	-3.39158774028762	35.61743539796412	p
-3.70957277553217	-10.90239364717474	34.54846883289474	c
-4.06859658744867	-10.95172199805313	37.22004973557403	c
4.85036473658244	-11.67450426680358	33.95905263829671	c
6.44886934846603	-8.58222638305124	36.52383994391185	c
-1.10166714229029	-13.94488525190905	36.31048985667746	c
4.68172829288408	-9.34990311324393	32.59909059395861	c
-1.84739010955547	-12.80684795506860	34.02477629551684	c
5.71220392242357	-7.44644323236912	34.23726954788186	c
5.90870260232729	-11.23996999219790	36.40278232711186	c
-2.46609252919086	-12.79866201237409	38.35754792581283	c
3.00817015008546	-3.07840887815440	38.83696742955433	p
-2.01068285879265	-6.01415301145599	41.50289854765375	p
-3.16408618925838	-4.11789732171250	37.72282267336816	p
-5.73427939834859	-9.16580666820727	32.23762086643680	si
-5.37541368184428	-9.74334502186632	38.24784705262120	h
4.23064967334519	-13.50620458988286	33.24877050176982	h
7.25696684299387	-7.59480203576101	38.13606568643168	h
0.26705498069986	-15.46951492396359	36.48026733128875	h
3.91648013085851	-8.81883805389604	29.20723284414845	si

-1.14242895468529	-13.32163950689858	32.16334210300999	h
5.86327902284546	-5.44289755054199	33.80098919610533	h
7.01707498330954	-13.57773690548313	38.78286061778454	si
-2.64286437736377	-13.88466742847017	41.70831730920352	si
1.04037043300335	-0.33359835275316	41.35072968714829	p
-2.88817315804054	-1.87338026443348	41.33269371539258	p
-6.77219978842210	-11.51692616238756	29.73642290869176	c
-8.58101869315042	-7.88963379833249	33.95581997090352	c
-4.12706778565422	-6.47731438332609	30.53555168608507	c
1.37463794625263	-11.05134978066317	28.05632923819410	c
2.93939432994527	-5.43400078991594	28.71521462691193	c
6.83922395544613	-9.46619077877834	27.23520916516195	c
4.88620165819134	-16.44159473639885	38.93814822065720	c
7.25844482539842	-12.01832917209066	41.97329713878117	c
10.26960706910921	-14.75568670988885	37.84233075364101	c
0.15188997815430	-12.99880067500363	43.73832397931918	c
-2.82519449858702	-17.46583803913598	41.67702872769686	c
-5.58880621830161	-12.53625920636374	43.18834102802702	c
0.18213000536944	2.84821142954518	38.60162163503404	p
-5.12115642038681	-12.28336515782243	28.72896424063033	h
-8.01815031022549	-10.62055555396887	28.33104368061197	h
-7.78769029043277	-13.11493200065964	30.59463168035639	h
-9.62510075067006	-9.40959359147437	34.91492183483841	h
-9.87512949986788	-6.92528473601514	32.64364166545449	h
-7.97122139874094	-6.51143055932266	35.38880475178719	h
-3.47701686365007	-5.02378588428122	31.86944516094007	h
-5.44800144476280	-5.58374900752109	29.19708605781026	h
-2.47499682295820	-7.11957130847845	29.45450042913475	h

1.89492347639188	-13.02737840133335	28.44361508943425	h
1.09595319575699	-10.85187797727299	26.00432702768796	h
-0.44135118789106	-10.68051298953845	28.98724615599915	h
1.45514284665013	-4.87605680840810	30.05801995376208	h
2.23850186731222	-5.11524979596153	26.78314127782520	h
4.55224523378489	-4.15987718243759	29.02839207012590	h
8.40708279325582	-8.24416402638136	27.84293972924216	h
6.48315331418593	-9.11264750981565	25.21503375239032	h
7.45257250746642	-11.44123100776469	27.45343424705095	h
2.99802786082874	-15.94743441421054	39.63966997783942	h
5.68577573547576	-17.88378758315295	40.20668122967228	h
4.66016332464921	-17.29755856920580	37.05631372767957	h
8.78665286614327	-10.60843188677105	41.97587695397088	h
7.66390202567840	-13.40491264241244	43.46974757824838	h
5.50308488350560	-11.01589023360650	42.45509262790784	h
10.20167465220183	-15.70524039970575	35.99351997826004	h
11.02187325531209	-16.10219050140320	39.24029258573674	h
11.59897331627651	-13.16500851390913	37.68902685706751	h
1.92777264141514	-13.77956624428086	42.99874692284529	h
-0.10341257798990	-13.73058527334463	45.67094507146982	h
0.37443921305398	-10.93635657266370	43.85059979509990	h
-4.46634874860147	-18.11405603742295	40.57805595717579	h
-2.97864726271694	-18.23035346464896	43.60657351554429	h
-1.11940530038340	-18.27500931362445	40.80319942526724	h
-5.46291370467006	-10.46063601766311	43.22466242473165	h
-5.81588320570069	-13.20470797735840	45.14508016678155	h
-7.28338494368531	-13.06740899452805	42.10843701045798	h
-1.19530196857308	5.81810457482428	41.25059823842548	p

4.19568058525522	4.02966290047013	38.04216893193077	p
-0.46791552309006	8.82923672411842	38.38331807180452	p
1.93900417821813	6.05122080448743	44.05268590713204	p
3.60747896293836	8.21758726120108	37.77900417021483	p
5.12894370833215	6.38823566440746	41.46182499679097	p
1.56755563602346	11.54520032962300	42.88038073934231	la
6.66804187640808	13.20356863459590	44.16979384134584	c
6.66924001978675	13.10526564111487	41.47558741991991	c
-1.62797302993163	14.78703811900833	45.83100838086462	c
-3.82155330304543	11.74768607764698	43.67578186093314	c
4.11342240519174	16.39138499120653	42.59700214664077	c
-1.47631339606619	12.51895399604947	47.28442455460097	c
5.05996972056418	15.28995820137039	44.82540769558427	c
-2.88073274159431	10.64957382126774	45.90500051639187	c
-3.04243758561743	14.34647436246212	43.57612299549089	c
5.09043779318546	15.03914385450993	40.45607176997503	c
8.83223865896326	11.39462909141325	46.29056318840343	si
7.72090748849168	11.74067823047089	40.35488946094983	h
-0.76077289232765	16.58228275090214	46.34953982269092	h
-4.92205117791135	10.76967444001499	42.24045134684059	h
2.86790157250851	18.02515522131214	42.52307073171216	h
-0.30720364848001	12.07140180085312	50.57095711857579	si
4.65580026255408	15.94575852027424	46.73152207305242	h
-3.14131714154137	8.68935318441689	46.46807145095676	h
-4.25341618049678	16.67378755114179	41.23623630057215	si
4.90980342034708	15.95960484735827	37.05648562564770	si
10.38749008553171	13.75282158937764	48.49984058664675	c
11.30580551580324	9.79127712475481	44.29229712108150	c

7.24874442926039	8.94074005279194	48.33530191218666	c
2.54927781082597	14.10643483406459	51.25459816429610	c
0.42916401158102	8.63468187859150	51.12181274815694	c
-2.86889070957580	13.07549516398170	52.86937682851107	c
-1.92602500302747	19.32147913699880	40.65447641953232	c
-5.04899129509349	15.01192740626397	38.19106553021842	c
-7.23873735794194	18.18466916927700	42.52369005250697	c
1.79967422616002	15.25293873631962	35.46102689183627	c
5.40582908938286	19.50615817869977	36.86804411857426	c
7.50126120073052	14.28115685203370	35.28079108211280	c
8.95486449252260	14.71227696158259	49.66413286211484	h
11.72864556988212	12.81241563229606	49.78356215467903	h
11.41134413790739	15.21052542566919	47.42827281745701	h
12.33877682028520	11.16685091109764	43.12564342085158	h
12.67617850750375	8.76894878579603	45.47694886950293	h
10.39564204891352	8.41508963912723	43.02644483933224	h
6.31846519373799	7.48586735304055	47.18101976667968	h
8.65567557586407	8.00254116827944	49.54986812463757	h
5.80580616007055	9.77536666024026	49.57306838712309	h
2.15395394733864	16.10289572783957	50.82644278822728	h
3.08155018531786	13.97427646647302	53.26144741947910	h
4.18581354301908	13.52737802328958	50.11969429533053	h
1.65979595429180	7.87975650415123	49.62733611529718	h
1.35866972162593	8.33748768779778	52.95848625778979	h
-1.31731379241536	7.50661121151781	51.09912555557752	h
-4.60525063256533	11.98069330206318	52.54155691732796	h
-2.27461693737707	12.77960973090879	54.84226663924503	h
-3.33153838194194	15.08789542988932	52.62268992933494	h

-0.20083207460202	18.62959223221658	39.73445572879224	h
-2.76530749349694	20.78031921449180	39.43142350659522	h
-1.37240973804728	20.22586854222874	42.44349921805831	h
-6.68241385434087	13.75337402244982	38.45786063480548	h
-5.52303109100670	16.36650337374641	36.68550991211301	h
-3.46703886799038	13.82896578307140	37.54570817414527	h
-6.84301177418293	19.20215419897744	44.29358567126004	h
-8.05295882377024	19.53377699090619	41.16345854032662	h
-8.66690133729876	16.73111809644609	42.93422236461766	h
0.21680587943582	16.23784064500797	36.37479571029814	h
1.85797299558266	15.86181441827591	33.47072655609453	h
1.38016603848800	13.21867049823623	35.50339065176655	h
7.23097930343294	20.04341182284368	37.70561650343068	h
5.36306152481418	20.17041942137434	34.89621373691956	h
3.91138977815348	20.50819898915187	37.91238153872534	h
7.19245728912820	12.22661733559971	35.37234174800045	h
7.52052945421378	14.84335936595601	33.27810260482326	h
9.36534914232624	14.69693718846121	36.10068066082604	h

Cartesian Coordinates of P_7^{3-} (given in atomic units)

2.57621555910170	0.00000000000000	0.06342373653848	p
-1.28810777955088	2.23106811980681	0.06342373653848	p
-1.28810777955088	-2.23106811980681	0.06342373653848	p
3.80582121145943	0.00000000000000	-3.82780710372043	p
-1.90291060572970	3.29593785138552	-3.82780710372043	p
-1.90291060572970	-3.29593785138552	-3.82780710372043	p
-0.00000000000000	0.00000000000000	-5.70684989845420	p

9. References

[1] M. C. Cassani, D. J. Duncalf, M. F. Lappert, *J. Am. Chem. Soc.* **1998**, *120*, 12958-12959.

[2] N. Reinfandt, C. Schoo, L. Dütsch, R. Köppe, S. N. Konchenko, M. Scheer, P. W. Roesky, *Chem. Eur. J.* **2021**, *27*, 3974-3978.

[3] C. T. Palumbo, L. E. Darago, M. T. Dumas, J. W. Ziller, J. R. Long, W. J. Evans, *Organometallics* **2018**, *37*, 3322-3331.

[4] G. M. Sheldrick, *Acta Crystallogr. Sect. A* **2015**, *71*, 3-8.

[5] G. M. Sheldrick, *Acta Crystallogr. Sect. C: Cryst. Struct. Commun.* **2015**, *71*, 3-8.

[6] O. V. Dolomanov, L. J. Bourhis, R. J. Gildea, J. A. K. Howard, H. Puschmann, *J. Appl. Crystallogr.* **2009**, *42*, 339-341.

[7] R. Ahlrichs, M. Bär, M. Häser, H. Horn, C. Kölmel, *Chem. Phys. Lett.* **1989**, *162*, 165-169.

[8] O. Treutler, R. Ahlrichs, *J. Chem. Phys.* **1995**, *102*, 346-354.

[9] A. D. Becke, *Phys. Rev. A* **1988**, *38*, 3098-3100.

[10] J. P. Perdew, *Phys. Rev. B* **1986**, *33*, 8822-8824.

[11] J. P. Perdew, *Phys. Rev. B* **1986**, *34*, 7406-7406.

[12] S. Grimme, J. Antony, S. Ehrlich, H. Krieg, *J. Chem. Phys.* **2010**, *132*.

[13] C. Ehrhardt, R. Ahlrichs, *Theor. Chim. Acta* **1985**, *68*, 231-245.

[14] J. Pipek, P. G. Mezey, *J. Chem. Phys.* **1989**, *90*, 4916-4926.
